# Guidelines for Micro–Computed Tomography Analysis of Rodent Dentoalveolar Tissues

**DOI:** 10.1002/jbm4.10474

**Published:** 2021-03-03

**Authors:** Michael B Chavez, Emily Y Chu, Vardit Kram, Luis F de Castro, Martha J Somerman, Brian L Foster

**Affiliations:** ^1^ Division of Biosciences, College of Dentistry The Ohio State University Columbus OH USA; ^2^ National Institute of Arthritis and Musculoskeletal and Skin Diseases (NIAMS) National Institutes of Health (NIH) Bethesda MD USA; ^3^ National Institute of Dental and Craniofacial Research (NIDCR)National Institutes of Health (NIH) Bethesda MD USA

**Keywords:** BIOMINERALIZATION, BONE, DENTAL CEMENTUM, DENTAL ENAMEL, DENTIN, ODONTOGENESIS

## Abstract

Micro–computed tomography (μCT) has become essential for analysis of mineralized as well as nonmineralized tissues and is therefore widely applicable in the life sciences. However, lack of standardized approaches and protocols for scanning, analyzing, and reporting data often makes it difficult to understand exactly how analyses were performed, how to interpret results, and if findings can be broadly compared with other models and studies. This problem is compounded in analysis of the dentoalveolar complex by the presence of four distinct mineralized tissues: enamel, dentin, cementum, and alveolar bone. Furthermore, these hard tissues interface with adjacent soft tissues, the dental pulp and periodontal ligament (PDL), making for a complex organ. Drawing on others' and our own experience analyzing rodent dentoalveolar tissues by μCT, we introduce techniques to successfully analyze dentoalveolar tissues with similar or disparate compositions, densities, and morphological characteristics. Our goal is to provide practical guidelines for μCT analysis of rodent dentoalveolar tissues, including approaches to optimize scan parameters (filters, voltage, voxel size, and integration time), reproducibly orient samples, define regions and volumes of interest, segment and subdivide tissues, interpret findings, and report methods and results. We include illustrative examples of analyses performed on genetically engineered mouse models with phenotypes in enamel, dentin, cementum, and alveolar bone. The recommendations are designed to increase transparency and reproducibility, promote best practices, and provide a basic framework to apply μCT analysis to the dentoalveolar complex that can also be extrapolated to a variety of other tissues of the body. © 2021 The Authors. *JBMR Plus* published by Wiley Periodicals LLC. on behalf of American Society for Bone and Mineral Research.

## Graphical abstract

Micro‐computed tomography (microCT) has become essential for analysis of mineralized tissues. Lack of standardized approaches and protocols for scanning, analyzing, and reporting data makes it difficult to understand how analyses were performed and reported, a problem compounded in the dentoalveolar complex by the presence of four distinct mineralized tissues: enamel, dentin, cementum, and alveolar bone. We aim to provide practical guidelines for microCT analysis, including how to optimize scan parameters (filters, voltage, voxel size, and integration time), reproducibly orient samples, define regions and volumes of interest, segment and subdivide tissues, interpret findings, and report methods and results.

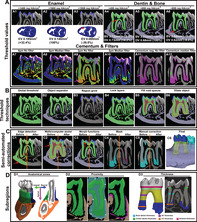

## Introduction

Micro–computed tomography (μCT) analysis has evolved into the gold standard for evaluating mineralized tissue microarchitecture in rodent research models, complementing and in some respects surpassing the capabilities of traditional histomorphometry by yielding data on two‐dimensional (2D) and three‐dimensional (3D) morphology, volume, and mineral density of skeletal elements.^(^ ^1^, ^2^, ^3^, ^4^, ^5^, ^6^, ^7^, ^8^ ^)^ The nondestructive approach, relatively short turnaround time, high throughput, and volumetric analyses are particularly appealing for those researchers analyzing bone cortical and trabecular architecture. In the early years of μCT analysis in skeletal research, lack of consistent methods and reporting made it difficult to critique analytical approaches, interpret some findings, and compare results across multiple studies. A timely publication in 2010 articulated specific and practical guidelines to standardize scanning parameters, analysis approaches, and reporting nomenclature for long bone (ie, typically femur or tibia) μCT analysis.^(^ ^1^ ^)^ That work has become a resource for those learning skeletal μCT analysis, planning μCT‐based studies, and citing standard methods.

As μCT use has expanded, analyses have grown to encompass complex structures featuring mineralized and nonmineralized tissues. An example is the dentoalveolar complex, which is unique and challenging to analyze because it features four distinct, sometimes contiguous, mineralized tissues: enamel, dentin, cementum, and alveolar bone. These hard tissues interface with adjacent soft tissues, the dental pulp and periodontal ligament (PDL), adding further complications. Some publications have focused on applications of μCT in the craniofacial region or oral cavity^(^ ^3^, ^9^, ^10^, ^11^, ^12^, ^13^, ^14^, ^15^ ^)^; though, to our knowledge only one publication has addressed more specific protocols for application in odontogenesis studies.^(^ ^16^ ^)^ However, despite current advances, the lack of standardized approaches for scanning, analyzing, and reporting data makes it difficult to understand how analyses were performed in many projects, how results should be interpreted, and if findings can be broadly compared to other models and studies, as well as making entry into μCT analyses challenging for researchers who are novices in the approach. We review considerations associated with analyzing the dentoalveolar complex, which is composed of tissues with similar and disparate compositions, densities, and morphological characteristics. To address these challenging complexities, we introduce innovative techniques that can also be extrapolated to other areas of the body.

Our goal in this report is to provide detailed and practical guidelines for μCT analysis of the dentoalveolar complex in order to increase transparency and reproducibility, promote best practices, and provide a basic framework for researchers who may benefit from such considerations. Using illustrative examples, we include a discussion of how to optimize scan parameters, reproducibly reorient samples, define regions and volumes of interest, segment and subdivide dentoalveolar tissues, interpret findings, and report methods and results. We focus on *Mus musculus* because of the many preclinical advantages, including ease in testing of therapeutic agents, reduced experimental time, flexibility in genetic manipulation, availability of established challenge and wound healing models, and accessibility of molecular reagents targeting murine models. Furthermore, the μCT concepts presented here can be broadly applied to large animal models and human cone beam computed tomography (CBCT) imaging.^(^ ^2^, ^17^, ^18^, ^19^, ^20^, ^21^, ^22^, ^23^ ^)^ We rely on previous publications^(^ ^1^, ^14^, ^16^ ^)^ to supply detailed technical explanations of concepts in order to focus on recommendations tailored to special complexities and challenges featured in dentoalveolar tissues.

## Overview of the Dentoalveolar Complex

**Fig 1:**
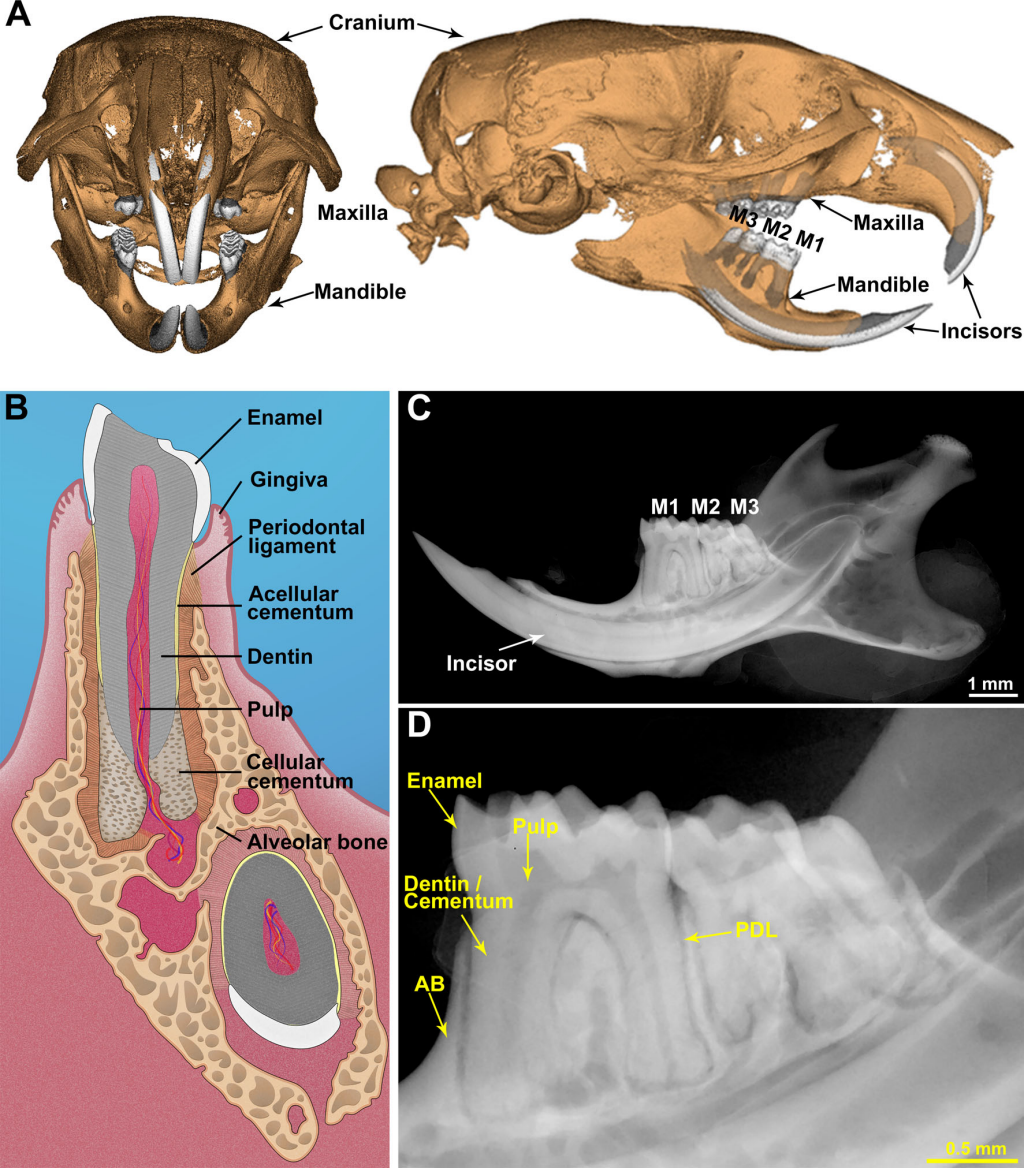
Murine dentoalveolar anatomy. (*A*) 3D μCT reconstruction of murine skull highlighting key landmarks including mandibular molars (M1–M3). (*B*) Schematic of mouse molar, incisor, and surrounding structures (coronal plane). (*C*,*D*) Radiograph of 90 days postnatal murine mandible highlighting dentoalveolar structures distinguishable by density and X‐ray absorbance: enamel, dentin/cementum, AB, and radiolucent unmineralized tissues including the PDL and pulp. AB = alveolar bone; PDL = periodontal ligament.

In this report, we focus on analysis of the mouse dentoalveolar complex, which includes the teeth and their supporting periodontal structures. We focus on the rodent mandibular first molar, the most commonly analyzed tooth and a reasonable model for human tooth development.^(^ ^24^, ^25^, ^26^ ^)^ Mice have a dental pattern of 1 | 3 in each quadrant, representing one (continuously erupting) incisor, a large diastema (toothless region), and three molars (Fig. 1*A*). As in human teeth, the mouse molar is divided into the crown, that portion visible above the gingiva, and roots that extend into the sockets formed by the maxilla or mandible. Four mineralized tissues are found in the dentoalveolar complex: enamel, dentin, cementum, and alveolar bone (Fig. 1*B*). Other anatomical locations in the mouse craniofacial complex are amenable to μCT analysis, but will not be featured in this report, including the continually erupting incisors, condyle and temporomandibular joint, basal bone not directly associated with the dentition, and the remainder of the cranium.

### Enamel

Enamel is the hardest and the most highly mineralized substance in the body, composed of >95% mineral by weight.^(^ ^9^, ^27^, ^28^, ^29^ ^)^ Enamel comprises the outermost layer of the tooth crown. During amelogenesis, the initial organic matrix deposited by ameloblasts achieves its full thickness and hydroxyapatite ribbons thicken and replace protein content.^(^ ^27^ ^)^ In its final mineralized state, ameloblasts are no longer present. Unlike humans, in which the enamel covers the entire crown, in rodents, enamel only covers part of the molar crown. Clearly visible on dental radiographs due to its radiopacity associated with its extremely high mineral content (Fig. 1*C*,*D*), the borders of fully mature enamel are also easily identifiable on μCT scans.

### Dentin

Dentin forms the bulk of the tooth crown and root, lies under the enamel, and surrounds the pulp chamber.^(^ ^30^ ^)^ Dentin is composed of a network of radiating tubules created by odontoblasts that lie at the dentin‐pulp border. Dentin is approximately 70% mineral by weight, however mineral content changes with location within dentin, age, maturation, and disease processes.^(^ ^30^, ^31^, ^32^ ^)^ Dentin is easily differentiated from enamel by radiograph due to relatively lower mineral content (Fig. 1*C*,*D*). Dentin encloses the pulp chamber that includes unmineralized collagen matrix, cells, blood vessels, and nerves.

### Cementum

Cementum envelopes the root dentin and is essential for anchoring PDL collagen fibers to the tooth, providing attachment and mechanical stability.^(^ ^33^, ^34^, ^35^ ^)^ Cementum is found primarily in two forms: acellular cementum covers the cervical portions of roots, and cellular cementum is localized to apical portions of roots and includes osteocyte‐like cementocytes. Cementum is approximately 45% to 50% mineral by weight, slightly less mineralized than dentin. Their adjacent position and similar mineral content make dentin and cementum difficult to differentiate in radiographs (Fig. 1*C*,*D*). On the exterior, cementum is surrounded by unmineralized PDL, and therefore the cementum‐PDL border is easily defined.

### Alveolar bone

The tooth‐associated bone that forms the sockets is referred to as alveolar bone, distinct from underlying basal bone of the maxilla or mandible (Fig. 1*C*,*D*).^(^ ^36^ ^)^ Like cementum on the root surface, PDL fibers insert into the bundle bone layer as Sharpey's fibers. Bone mineral density can vary widely by site, age, maturation, and other factors; however, on average, bone is estimated to be 50% to 60% mineral by weight.^(^ ^36^, ^37^ ^)^ As bone is separated from dental tissues by the unmineralized PDL, it is easily distinguished from the other three hard tissues. The cementum‐PDL‐alveolar bone (along with gingiva) forms the periodontal complex that attaches and supports the tooth.^(^ ^38^ ^)^

## Image Acquisition of Dentoalveolar Tissues

**Fig 2:**
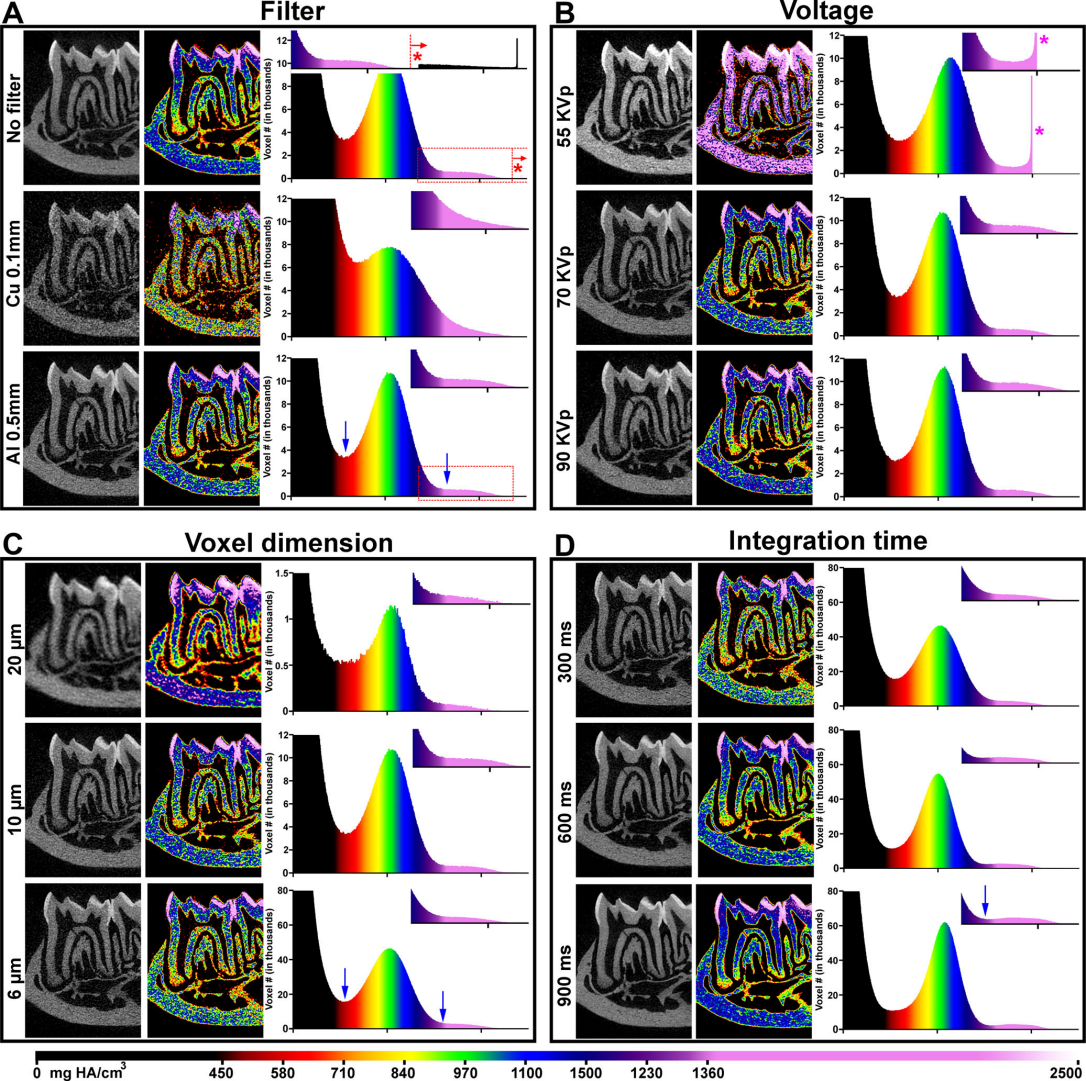
Optimization of μCT scan parameters for dentoalveolar structures. All panels depict the same 6‐week‐old mouse mandible that has been scanned and calibrated. Each optimization scan includes a grayscale image, a heat map of densities, and a density histogram reporting the number of voxels detected in the scan. Inserts in histogram panels show a higher magnification of densities corresponding to enamel‐like densities. Color scale for heat maps and histograms is at bottom of figure. (*A*) To test the effects of prismatic filters, scans were performed with no filter, 0.5‐mm Al filter, or 0.1‐mm Cu filter. (*B*) To test effects of voltage, scans were performed with 55, 70, or 90 kVp. (*C*) To test the effects of resolution, scans were performed at voxel size of 20, 10, or 6 μm. (*D*) To test the effects of integration time, scans were performed at 300, 600, or 900 ms. Discussion of scanning results and optimization of parameters are described in the text.

Because the dentoalveolar complex features four distinct mineralized tissues with different compositions, it is imperative to optimize μCT scanning parameters for assessment of tissue‐specific effects resulting from modulation of genes, proteins, environment, trauma, aging, etc. Optimal parameters to visualize one tissue may not work well for others because of differences in mineral densities. Numerous reviews have described in detail the concepts underlying μCT scanning^(^ ^1^, ^5^, ^6^, ^14^, ^18^, ^22^, ^39^, ^40^, ^41^, ^42^ ^)^; therefore, we will introduce these only briefly in order to focus on specific challenges related to scanning the dentoalveolar complex. There are several approaches for sample preparation; however, for results shown here we used a standard method employing formalin‐fixed tissues scanned under aqueous conditions in 70% ethanol, in order to maintain samples at biologically relevant levels of hydration and avoid cracking of mineralized tissues. There are a variety of μCT scanners available for purchase or use in core facilities. All results included here are obtained from a Scanco μCT 50 ex vivo cabinet system (Scanco Medical, Brüttisellen, Switzerland). Each scanner model will feature different capabilities and limitations (eg, resolution, filters, space dictating size of analyzed samples, density capacities, and fields of view), and therefore will require its own optimization for dentoalveolar tissues. However, the optimization steps outlined below can be adapted to all scanners based on our detailed evaluation criteria of scan results shown in Fig. 2. There are numerous options for analysis software, including proprietary applications sold with scanners.^(^ ^16^, ^18^, ^40^, ^42^, ^43^ ^)^ These also have their own capabilities and limitations that dictate to some degree the ease and efficiency of analysis. All analyses and images included in this report were accomplished using Scanco proprietary software for scan reconstructions (default settings) and AnalyzePro 1.0 medical imaging software (Analyze Direct, Overland Park, KS), a software system used in our studies.^(^ ^23^, ^44^, ^45^, ^46^, ^47^, ^48^, ^49^ ^)^

CT images are created using X‐rays directed and rotated around a sample (or a sample rotating in relation to the X‐ray source), creating a series of projections. X‐rays are a form of electromagnetic radiation with high photon energy, and image generation relies on the partial transmission of X‐rays; ie, differential absorption of X‐rays by tissues. Based on previous reports, common scanning parameters optimized for image acquisition are: inclusion of a filter, voltage, voxel size, and integration time, all of which alter the absorption of X‐rays by a sample.^(^ ^1^, ^4^, ^5^, ^6^, ^16^, ^18^, ^22^, ^40^, ^41^, ^42^, ^50^, ^51^ ^)^ In order to compare images acquired from different scanning parameters, we scanned the same 6‐week‐old mouse mandible, systematically varying filter, voltage, voxel size, and integration time (Fig. 2, Table 1). Scans were calibrated against a set of five hydroxyapatite (HA) phantoms of known density (0, 100, 200, 400, and 800 mg HA/cm^3^) scanned with the same parameters. This density calibration using calibration phantoms is routine and essential for quantitative μCT, mimicking the attenuation of different tissue types and densities, and providing a standard curve to calculate absolute or relative density units for samples, allowing cross‐study comparisons and helping detect and control for potential changes in the scanner X‐ray source over time. Calibration phantoms are often composed of resin‐embedded HA. Although the μCT scanner measures only X‐ray attenuation and does not identify HA per se, HA is the major mineral component in bones and teeth. Thus, the assumption is made that attenuation from vertebrate skeletal and dental materials will match that of HA phantoms in linear fashion (in some situations, the nature of the mineral may need to be confirmed by additional techniques such as spectroscopy or electron microscopy). Importantly, calibration curves have been validated even for highly mineralized enamel.^(^ ^9^, ^52^, ^53^ ^)^ The limitations of density calibration phantoms, their correlation to tissue mineral content, and other factors have been tested and discussed in detail elsewhere, and these sources can be consulted for more detailed information on the subject.^(^ ^1^, ^2^, ^8^, ^9^, ^54^, ^55^, ^56^, ^57^, ^58^, ^59^, ^60^ ^)^ Geometric calibration of the μCT scanner is also critical for proper reconstruction of 2D sample data, reduction of scan artifacts, and optimization of resolution. Scanners typically have a geometric calibration protocol that should be operated on a regular schedule, and additional references can be consulted for more information on this topic.^(^ ^2^, ^61^, ^62^ ^)^

**Table 1:** Selection of μCT Scan Parameters

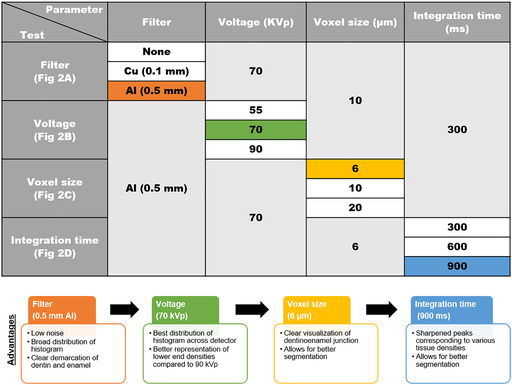

Variables including filter, voltage, voxel size, and integration time were optimized for murine dentoalveolar tissues.

Here we illustrate the effects of changing scanning parameters in optimization scans of mouse mandibles by showing X‐ray absorption as a grayscale image, a density heat map, and a voxel frequency distribution in mg HA/cm^3^. Each reconstructed grayscale image is composed of millions of voxels, which vary depending on scan parameters. Heat maps were generated to demonstrate density distributions, with 500 mg HA/cm^3^ corresponding to red and 1600 mg HA/cm^3^ to lilac (Fig. 2). When counts assigned to each value (in mg HA/cm^3^) are plotted on a frequency graph, an overwhelming number of voxels are considered background noise. Each frequency graph depicts two main peaks: the taller peak at a lower density range corresponds to dentin/bone/cementum, and the shorter peak at a higher density range corresponds to enamel (top right of each graph in Fig. 2). To optimize scanning parameters, images were evaluated based on the amount of noise, contrast between mineralized tissues, and clarity of structures.

### Filter

Physical filters can be applied during μCT scans, and by concentrating the X‐ray beam, they act to reduce noise and increase contrast. Additionally, applying a filter can reduce artifacts caused by low energy X‐rays.^(^ ^1^, ^18^, ^63^, ^64^ ^)^ The strength of the filter (ie, composition and thickness) should be considered during selection; stronger filters are indicated for denser samples; eg, mineralized tissues. Holding the voltage (70 kVp), voxel size (10 μm), and integration time (300 ms) constant, we compared no filter to a 0.1‐mm copper (Cu) filter or 0.5‐mm aluminum (Al) filter (Fig. 2 *A*, Table 1). The scan with no filter appeared relatively crisp in grayscale; however, the heat map revealed numerous red and orange (low density) voxels and very poor separation of crown dentin and enamel. This is shown in the histogram as an expanded single peak where the enamel peak has been absorbed into the dentin/bone/cementum peak that skewed right (Fig. 2*A*, red *). There are also numerous artifacts of very high‐density voxels well above biologic density values.

The scan with the Cu filter did not show any improvement in dentin and enamel separation and distribution of voxel densities, which was exacerbated by an unacceptable amount of noise in the grayscale and heat map images. The heat map and flattening of peaks in the histogram indicated that the noise holds density values similar to those of dentoalveolar tissues, preventing digital filtering of noise from enamel, dentin, and cementum and rendering the scan unusable for analysis.

The scan using the 0.5‐mm Al filter yielded clearer images without excess noise, better distribution of voxel densities in the heat map, and better demarcation between enamel and dentin. The more compressed dentin/bone/cementum peak was better separated from the enamel peak (Fig. 2*A*, blue arrows). Because of these advantages, subsequent scans were performed with the 0.5‐mm Al filter.

### Voltage

Voltage corresponds to the energy of the photons passing through a sample and is expressed in kiloelectron volts (kEV). In μCT scanning, the X‐ray tube potential is specified by the user. Expressed in kVp, the X‐ray tube potential corresponds to the applied peak electron potential of the X‐ray tube that accelerates electrons for generating X‐ray photons.^(^ ^1^ ^)^ Generally, lower voltages provide better contrast between tissues, particularly for tissues with lower densities.^(^ ^1^, ^6^, ^18^, ^22^, ^40^, ^41^, ^42^ ^)^ However, voltages that are too low result in decreased transmission of X‐rays through the sample that reach the detector. In contrast, voltages that are too high lead to increased transmission of X‐rays through the sample that reach the detector, which can result in increased scatter and reduced resolution of less dense materials (i.e. decreased signal to noise ratio). For dentoalveolar tissues, the voltage would optimally be high enough to pass through enamel and minimize noise but low enough to generate contrast between tissues with similar densities; eg, cementum and dentin. Holding the filter (0.5‐mm Al), voxel size (10 μm), and integration time (300 ms) constant, we compared scan voltages of 55, 70, and 90 kVp (Fig. 2 *B*, Table 1). Changing kVp resulted in altered brightness and contrast in grayscale images and shifts in density values in the heat maps. The scan acquired with 55 kVp was not ideal for dentoalveolar tissues. The photon energy was too low and unable to penetrate high density tissues, leading to lower numbers of photons reaching the detector and a truncated histogram around 2000 mg HA/cm^3^ (Fig. 2*B*, pink *). With increasing voltage at 70 or 90 kVp, the dentin/cementum/bone peak narrowed, and the enamel peak emerged. Thus, 70 and 90 kVp scans both showed acceptable image contrast and enamel separation. A voltage of 70 kVp was used in subsequent scans due to acceptable enamel separation and better separation potential and detection of the lower end of density for dentin/cementum/bone.

### Voxel size

Voxel size is the size of a 3D pixel in the reconstructed image following μCT scanning. Smaller voxel sizes correspond to higher resolution scans. Smaller voxel sizes allow for the visualization of a greater number of structures in more detail and accuracy, particularly small features. However, in practical terms, smaller voxel sizes require increased time per scan and produce increased file sizes. Bouxsein and colleagues^(^ ^1^ ^)^ recommended that the minimum ratio of voxels to objects of interest should be 2 (eg, a minimum of two voxels per bone trabecula, thickness of cementum, or other feature), with higher ratios resulting in more accurate measurements.^(^ ^1^, ^16^, ^18^, ^50^, ^65^, ^66^, ^67^ ^)^ Holding the filter (0.5‐mm Al), scan voltage (70 kVp), and integration time (300 ms) constant, we compared voxel sizes of 20, 10, and 6 μm (Fig. 2 *C*, Table 1).

The grayscale image revealed that a 20‐μm voxel size was wholly inadequate for the mouse dentoalveolar complex, with substantial blurring and noise in the soft tissues of the pulp and PDL. The corresponding heat map depicted dentin as nearly homogenous in density, and the y axis of the frequency distributions showed that voxel size dramatically influenced the counts per density. Borders of enamel and dentin, tissues with significantly different densities, were poorly resolved in grayscale, heat maps, and on the histogram. In contrast, higher resolution (lower voxel size) resulted in a clearer dentinoenamel junction, enhanced contrast of structures (eg, we could begin to resolve cellular cementum from dentin), and visualization of much greater detail in bone structures. Smaller voxel sizes also resulted in higher counts and sharper dentin/cementum/bone and enamel peaks, more amenable for segmentation of the tissues from one another for analysis.

In the scanner we used (Scanco μCT 50), the voxel size is restricted by the sizes of the sample holders, enclosed tubes in which samples are loaded and can remain hydrated during scanning. Other scanners use alternative methods for sample stabilization during scanning.^(^ ^64^ ^)^ In Scanco scanners, smaller sample holders that reduce the distance between the X‐ray source, sample, and X‐ray detector, are capable of higher resolutions. To reduce scan time and file sizes, we optimized scans based on a 19.0‐mm (diameter) sample holder. This was the smallest sample holder that allowed for mandibles to be loaded where molar occlusal plane was perpendicular to sample holder length (ie, mandibles were laid on their sides and stacked in a column with each separated from the next by a foam spacer, efficiently allowing multiple mandibles to be scanned per sample holder). A smaller sample holder would require mouse mandibles to be loaded in a different orientation (increasing scanning time and file size further) or trimmed (requiring additional preparation with the possibility of damaging target tissues). According to the manufacturer's recommended settings, the smallest voxel size for the 19.0‐mm sample holder was 6 μm, which we have found to be adequate for dentoalveolar tissues (see below under Examples of Analyses). Because no substantial advantage for enamel, dentin, and bone was noted with smaller for the 2‐μm voxel setting, a voxel size of 6 μm was used in subsequent scans. There is one exception for segmentation of acellular cementum (described below under Examples of Analyses: Cementum and PDL) where a 6.0‐mm sample holder was necessary for 2‐μm voxel scans to accurately separate murine acellular cementum from dentin.

### Integration time

Integration time refers to the time spent on each projection (in milliseconds; ms), and along with frame averaging (the number of times each projection is repeated), can change the number of photons directed at the sample.^(^ ^1^, ^18^, ^51^ ^)^ Therefore, increasing integration time increases overall scan time, but does not increase file size. Holding the filter (0.5‐mm Al), scan voltage (70 kV), and voxel size (6 μm) constant, we compared integration times of 300, 600, and 900 ms (Fig. 2 *D*, Table 1).

In grayscale images, increasing integration time reduced noise in pulp and PDL soft tissues, and removed speckling in mineralized tissues. Although there are only subtle differences between the three integration times in the grayscale images, in heat maps and frequency distributions, dentin and cellular cementum were better differentiated by density when 900 ms integration time was used. Increased integration time did not shift locations of peaks in the frequency graphs, but instead sharpened the dentin/cementum/bone and enamel peaks. We did not attempt integration times greater than 900 ms for practical reasons. However, there is room for improvement, although the detector could potentially become saturated at some point, with no further benefit to increasing time.

### Summary of selection of scan parameters

In the previous sections under Image Acquisition of Dentoalveolar Tissues, we demonstrated the impact of filter, voltage, voxel size, and integration time on optimization of scan parameters, landing on 0.5‐mm Al filter, 70 kVp, 6 μm voxel size, and 900 ms integration time (Fig. 2; Table 1). Filter had the greatest impact on scans, with Cu filter and no filter resulting in unusable scans. Next, voltage dramatically altered density distributions and visualization of mineralized tissue; 70 kVp was determined to be the best for resolving structures within 500 to 1600 mg HA/cm^3^. For qualitative and quantitative assessments, 6 μm is suitable for separating enamel, bone, dentin, and cellular cementum (see subsequent sections). Last, we chose an integration time of 900 ms based on our goal to segment dentin and cellular cementum. Each one of the aforementioned parameters can alter quality of scans. At a minimum, the make and model of scanner, the filter, voltage, voxel size (resolution), and integration time should be reported in methods sections of manuscripts employing μCT.

## Image Processing

Tissues of the oral cavity present several unique challenges for μCT analysis in comparison to sites more frequently analyzed, eg, lumbar vertebrae or long bones, though their successful analysis shares a few key steps: orientation, region of interest (ROI), and segmentation.

### Orientation

**Fig 3:**
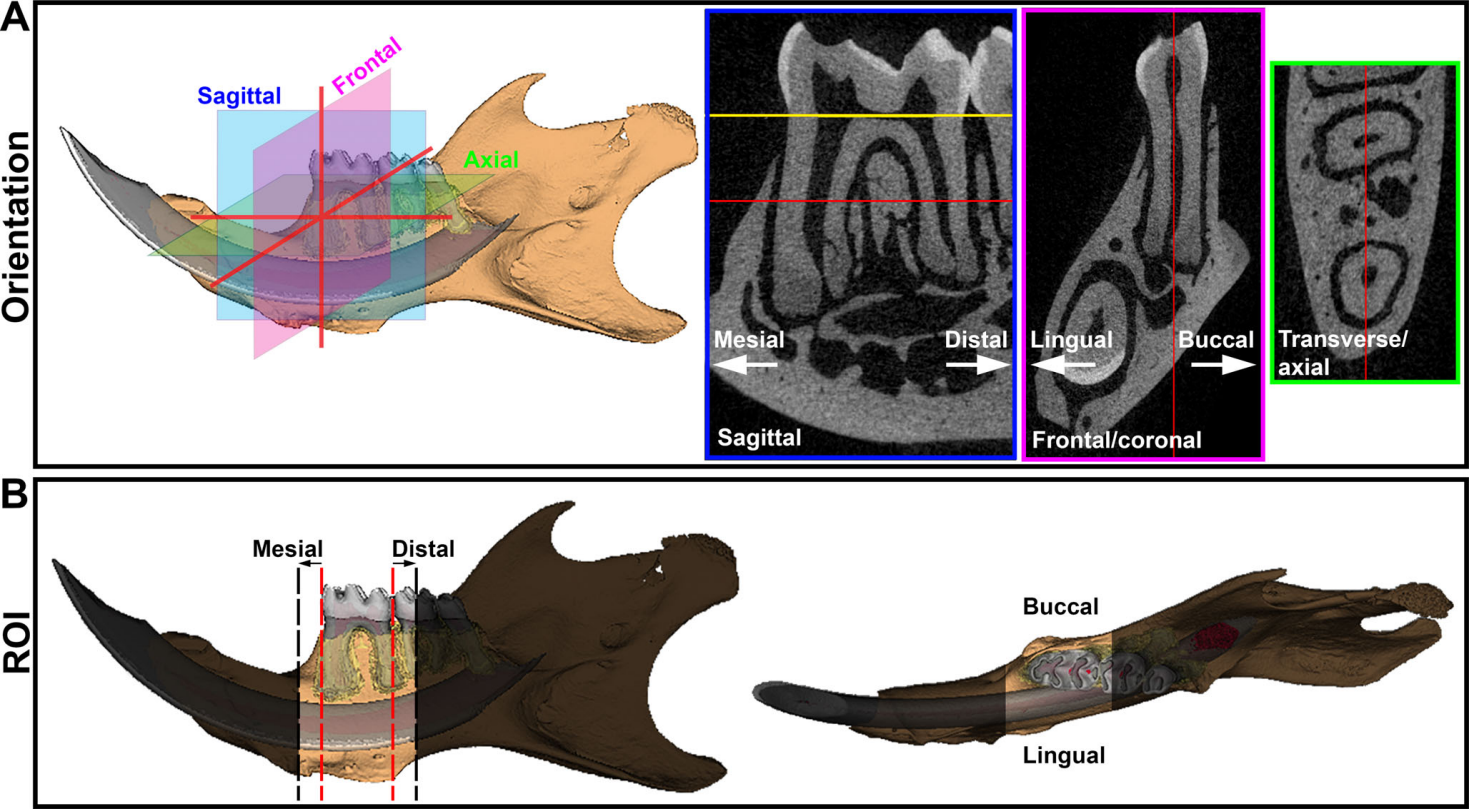
Orientation and ROIs in the dentoalveolar complex. (*A*) 3D and 2D representative images demonstrating schematic of sagittal, frontal/coronal, and transverse/axial planes anatomical planes for consistent orientation of mouse mandible. Red lines indicate intersecting planes for optimal orientation and consistency. (*B*) ROI was determined after mandible reorientation by finding the M1 mesial and distal edges (red dotted lines) and expanding the region 480 μm mesial and distal (black dotted lines) to include the alveolar bone in these regions. The shaded area indicates regions excluded from analysis. ROI = region of interest.

Digital orientation of samples after scanning is the first step toward reproducible results. This is true for femur and tibia analyses, where the long axis of the bone is used to realign the sample prior to selection of trabecular and cortical ROI.^(^ ^1^ ^)^ The mandible and maxilla are more complex shapes, and landmarks must be carefully chosen.^(^ ^16^ ^)^ Though orientation is often not described in detail, many papers reporting dentoalveolar analyses use one or more of the molars as the most consistent anatomical landmarks to orient mandibles/maxillae, as can be deduced from their figures.^(^ ^11^, ^13^, ^23^, ^44^, ^45^, ^48^, ^68^, ^69^, ^70^, ^71^, ^72^, ^73^, ^74^, ^75^ ^)^ Incisors or whole mandible/maxilla approaches are also sometimes used for orientation.^(^ ^9^, ^72^, ^76^, ^77^ ^)^ Here, we describe the use of the mandibular first molar (M1) as a guide for orientation. To achieve consistency, orientation in sagittal, frontal/coronal, and transverse/axial planes should be standardized across samples using anatomical landmarks. In the sagittal view, the plane generated by the mesial and distal aspects of the cementoenamel junction (CEJ) was oriented parallel to the transverse plane (Fig. 3*A*). In the transverse view, the plane generated by the center of the mesial and distal root canals of the first molar were oriented parallel to the sagittal plane. In the coronal view, the plane bisecting the pulp of the mesial root was oriented parallel to the sagittal plane. Although orientation is important for consistent imaging of M1, it becomes even more critical when measuring surrounding tissues like alveolar bone (as described in more detail below under Examples of Analyses: Alveolar Bone). This digital orientation approach yielded highly reproducible results during training of multiple users in our laboratories (<0.1% difference in volume measurements using the same mandible scan), validating this approach (data not shown). Experimental factors may dictate which teeth serve as landmarks, eg, if a ligature is placed on the second molar to induce periodontal breakdown, then the second molar may be a better choice as the central landmark for mandible orientation. When reporting μCT analysis of the mouse mandible, the approach to sample orientation should be described in enough detail and/or images should be included to illustrate the approach.^(^ ^69^ ^)^

### ROI

After orientation, the ROI is defined based on experimental questions to be answered. In long bones, there are prescribed ROIs to analyze trabecular and cortical bone based on anatomical landmarks (eg, proximal or distal growth plate) and recommended minimum numbers of slices or μm.^(^ ^1^, ^50^, ^78^ ^)^ For dentoalveolar analysis, the tooth is a well‐defined and self‐contained organ that can be analyzed in whole, an approach not usually taken for long bones. Due to limitations in software, computing power, and/or time, some investigators may opt to analyze more limited ROIs rather than the entire tooth. For example, rather than segmenting the entire dentin of the molar, a cubic, ring, or other shaped ROI of a certain number of voxels or μm^3^ may be chosen within dentin and used as a representative sample. Although this approach may be necessary in some circumstances, it should be avoided if possible and used with caution as it can bias results and provide density values that inaccurately represent the bulk tissue properties; eg, densities of the dentoalveolar tissues vary by location.^(^ ^79^ ^)^ If this restricted ROI approach is used, locations must be chosen stringently and consistently across samples to minimize selection bias. In terms of creating the ROI, more details on segmenting dental tissues from one another are discussed below under the Segmentation section.

Orientation and landmarks used to define the ROI must be anatomically similar across samples to capture potential differences between experimental and control groups. In our studies, the ROI was determined using a reoriented M1 and included all alveolar bone buccal and lingual to the molar and extending the region 480 μm forward from the mesial root and 480 μm backward to the distal root, using the most mesial and distal root locations, respectively (Fig. 3*B*). This ROI was selected to include the alveolar rise mesial to M1 and the interdental bone between M1 and M2, and this approach has been applied successfully to a wide range of mouse mandibles aged 2 weeks to over 1 year old.^(^ ^23^, ^44^, ^45^, ^47^, ^48^, ^49^, ^69^, ^80^, ^81^ ^)^ The ROI we describe here would suffice for many studies of alveolar bone but should be experimentally determined for the hypothesis being examined and the specific needs of the study. In publications, the ROI should be described in detail, including anatomical landmarks used and precise numbers of slices or μm for tissues like alveolar bone.

### Segmentation

Following orientation and ROI definition, segmentation is the next step toward establishing consistency in μCT analyses. Segmentation, or isolation of structures, typically begins with setting thresholds to separate tissues by mineral density.^(^ ^1^, ^50^, ^78^, ^82^ ^)^ Setting appropriate thresholds is imperative to promote accuracy and reproducibility, because measurements can be greatly influenced by the choice of threshold values. Thresholds for all target tissues should be defined in HA/mg, Hounsfield units, grayscale values, or similar absolute or relative units. Although relative units (eg, grayscale values) are adequate for comparisons within a given study (same scanner, settings, and short time span between scans), calibrated absolute units (eg, mg HA per volume and Hounsfield units) allow more robust comparisons between studies and compilation of normal and abnormal data sets across the published literature.^(^ ^1^, ^4^, ^5^, ^11^, ^13^, ^50^, ^56^ ^)^ As shown in Fig. 2, use of calibrated absolute units allows comparison of scans acquired from different sets of parameters.

**Fig 4:**
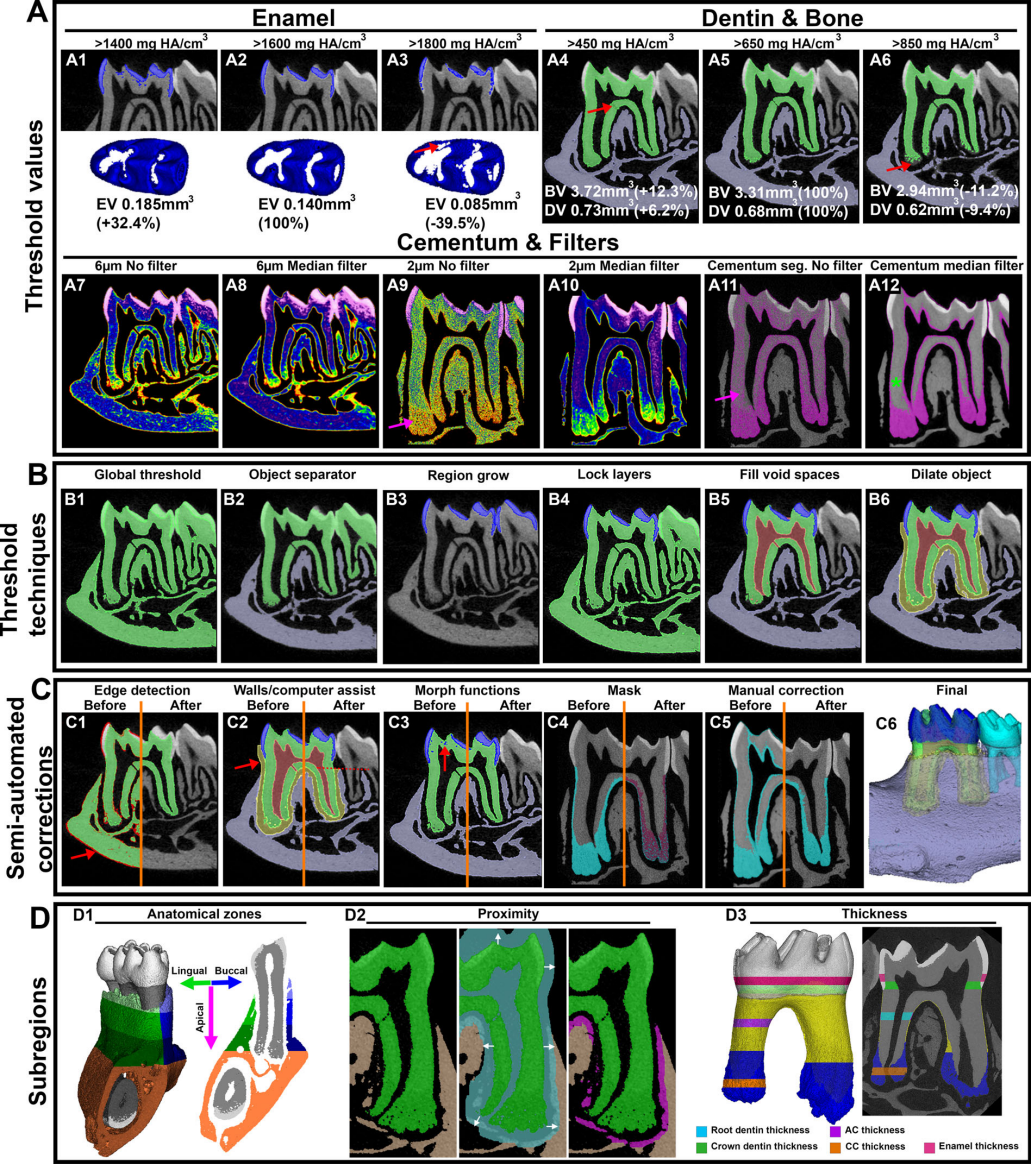
Strategies for segmentation of dentoalveolar tissues. (*A*1–*A*12) Selection of threshold values (mg HA/cm^3^) will affect calculation of EV, DV, and BV measurements. (*A*1–*A*6) Thresholds and resulting 2D images and calculated volumes are reported in the top row; (*A*7–*A*12) the second row demonstrates use of filters to accomplish segmentation of cementum from dentin, particularly how scan resolution and median filters can substantially improve fidelity of segmentation. Details are described in the text. (*B*1–*B*6) Threshold techniques are available to segment tissues quickly and reproducibly, including software operations: object separator, region grow, lock layers, fill void spaces, and dilate object. (*C*1–*C*6) Semi‐automation of corrections after segmentation is recommended to save time and increase reproducibility. These include: edge detection (software uses filters to identify borders; red arrow), walls (user sets boundaries to help software define borders; red arrow and red dotted line), morph functions (close function is highlighted, where software dilates then erodes the object to original position unless the dilated object was fully encompassed by the function, thus correcting small internal errors; red arrows), mask (software uses a previously generated mask, registers it to same position and allows for segmentation only within/outside the mask), and manual correction (here, we show removal of areas that do not anatomically correspond to cementum). (*D*1–*D*3) Subregions of tissues can be defined for targeted analysis. For example, mandibular bone is separated into basal bone and buccal and lingual aspects of alveolar bone. Proximity can be used to define areas within a certain anatomical structure, such as bone within a certain distance from the tooth (magenta). Thicknesses can be measured, such as in enamel, different regions of dentin, AC, and CC. AC = acellular cementum; BV = bone volume; CC = cellular cementum; DV = dentin volume; EV = enamel volume.

Recommendations for thresholding bone tissue in skeletal analysis have been discussed.^(^ ^1^ ^)^ However, the dentoalveolar complex offers the challenge of four unique mineralized tissues, requiring further considerations. Thresholding of dentoalveolar tissues is based on anatomical structures and inherent density differences between tissues. Because average enamel mineral density is considerably greater than dentin, bone, and cementum, simple thresholding is largely sufficient to segment enamel (Fig. 4*A*1–*A*3). The significance of threshold values can be appreciated using tissue volume as a readout. There is no absolute correct threshold for any of the tissues, and each researcher should optimize their thresholds according to their scanner and scan conditions. However, we provide examples of how to evaluate thresholds and how their choice directly affects enamel, dentin, and bone volumes calculated. Based on anatomical landmarks examined over several studies, 1600 mg HA/cm^3^ was determined to most accurately label enamel in our scans.^(^ ^44^, ^45^, ^46^, ^47^, ^48^, ^49^, ^69^, ^81^ ^)^ Qualitatively, a lower threshold (1400 mg HA/cm^3^) resulted in areas of dentin being identified as enamel (visualized as speckling in dentin areas), whereas a higher threshold (1800 mg HA/cm^3^) resulted in enamel volume (EV) underestimation (seen as visible gaps in 3D renderings, Fig. 4*A*3 red arrow). Quantitatively, these lower and higher thresholds resulted in substantially different EV values at +32.4% and −39.5%, respectively.

Thresholding for dentin and bone was similarly evaluated. We selected a lower threshold of 650 mg HA/cm^3^ as most accurately segmenting bone and dentin volumes (BV and DV, respectively) in our scans. A lower threshold (450 mg HA/cm^3^) included areas that did not anatomically correspond to dentin; for example, an accessory canal was mislabeled as dentin (Fig. 4*A*4, red arrow). Conversely, a higher threshold (850 mg HA/cm^3^) excluded apical regions of dentin (Fig. 4*A*5, red arrow). While these differences appear subtle in 2D images, the resultant volumetric changes were not insignificant. Quantitatively, the lower threshold resulted in values +12.3% in BV and + 6.2% in DV, and the higher threshold resulted in values −11.2% in BV and −9.4% in DV (Fig. 4*A*4–*A*6).

Because of their similar densities, cementum is particularly challenging to segment from dentin, but the two can be distinguished with appropriate resolution and image processing filters. Although acellular cementum in mice is extremely thin (less than 10 μm thickness at ages typically used, eg, up to 6 months postnatal), cellular cementum on the apical portions of roots grows rapidly after tooth eruption.^(^ ^35^, ^83^, ^84^ ^)^ In our experience, a high resolution (2 μm voxel size) and a high integration time (1200 ms) must be used in conjunction with proper filter and voltage to reliably segment mouse dentin from acellular cementum (Fig. 4*A*7–*A*12).^(^ ^23^, ^45^ ^)^ As seen in the 6‐μm and 2‐μm heat maps with no filter, the cementum layer has a lower average density than dentin (Fig. 4*A*7,*A*9, as indicated by red/orange/yellow voxels), which becomes clearer with higher resolutions (compare Fig. 4*A*7 to Fig. 4*A*9, pink arrow). However, a general threshold alone cannot properly differentiate the tissues as dentin also includes areas with lower density similar to cementum (Fig. 4*A*11, pink arrow). To further enhance native density differences, a median filter was applied, which resamples voxels to the median density of their neighbors (Fig. 4*A*12, green asterisk). This results in a clearer demarcation between dentin and cementum, allowing for improved segmentation. With the median filter, cellular cementum is readily viewed in 6‐μm and 2‐μm scans; however, 6 μm is inadequate for isolating acellular cementum. Even with the median filter in a 2‐μm scan, newly mineralized dentin immediately adjacent to pulp had similar density values to cementum (Fig. 4*A*12). Sequential steps are required to manually correct this situation, as outlined in the following paragraphs.

The thresholding values described here were optimized in normal adult mice, however, optimal thresholding values should be determined for the project and scientific question(s). For example, in some contexts (eg, very young mice, models of bone healing, or genetically modified mice with defective mineralization), a threshold value for alveolar bone at 650 mg HA/cm^3^ may exclude hypomineralized bone and report a low tissue volume even if there is expansion of poorly mineralized (osteoid) bone.^(^ ^49^ ^)^ Threshold values should always be included in publications and any limitations or alterations from the norm should be discussed.

After optimal threshold values are determined, a myriad of computer software‐based histometric thresholding techniques is available. The most familiar and least selective is a global threshold where one value is set for all tissues in a sample. Global thresholds are useful for separating out structures that are homogeneous in density or for separating out objects from background noise, but they are inadequate when numerous tissues present have overlapping densities; eg, dentin and bone (Fig. 4*B*1). With computer and software advances, new histometric thresholding techniques are available. For all segmentation procedures described in the following paragraphs and shown in Fig. 4B and 4C, we used semi‐automatic and manual tracing features in AnalyzePro to segment tissues quickly and reproducibly. Highlighted in Fig. 4*B* are useful software operations: object separator (Fig. 4*B*2; objects that are spatially distanced are separated), region grow (Fig. 4*B*3; object definition is based on defined threshold value and connected components), lock layers (Fig. 4*B*4; a segmented layer is locked from changes in subsequent steps), fill void spaces (Fig. 4*B*5; spaces enclosed within a segmented object are identified as a new object), and dilate object (Fig. 4*B*6; a labeled region is expanded by a user defined distance).

Although the majority of segmentation can be accurately achieved with threshold techniques illustrated in Fig. 4*B*, correction is required. Many semiautomatic correction tools are available, which save time and increase reproducibility, including: edge detection (Fig. 4*C*1; software uses filters to identify borders, red arrow), walls (Fig. 4*C*2; user sets boundaries to help software define borders; red arrow and red dotted line), morph functions (Fig. 4*C*3; close function is highlighted here where software dilates then erodes the object to original position unless the dilated object was fully encompassed by the function, thus correcting small internal errors, red arrows), mask (Fig. 4*C*4; software uses a previously generated mask, registers it to same position and allows for segmentation only within/outside the mask), and finally, manual correction, ie, non‐automatic, operator‐specific corrections based on previous anatomical knowledge (Fig. 4*C*5; here, we show removal of areas that do not anatomically correspond to cementum).

Our approach includes multiple sequential steps. For tissues with disparate densities, eg, dentin and enamel, region grow for a single threshold can be used (Fig. 4*B*3). Because region grow searches for connected components, high density voxels in the dentin are excluded when a seed point is set on enamel. Areas in dentin that are incorrectly assigned to enamel can be eliminated with software operations such as morphological transformations (Fig. 4*C*3). Once the enamel layer is defined, locking the layer allows a second threshold to be set for dentin/bone without altering the enamel layer (Fig. 4*B*4). For tissues with a similar density, semiautomatic segmentation requires anatomic spatial separation, eg, dentin and bone, which are separated by an unmineralized PDL that typically spans 50 to 100 μm in width^(^ ^47^, ^48^ ^)^ (Fig. 4*B*2). For contiguous tissues with similar densities (eg, dentin and cementum), additional processing methods can be employed. We applied a median filter with a kernel size of seven to reconstructed images better identified density differences between dentin and cementum (compare Fig. 4*A*10 to Fig. 4*A*12). A threshold between 250 to 1100 mg/cm^3^ was applied to capture all possible cementum tissue, with minor manual corrections to remove less dense dentin adjacent to the pulp chamber (Fig. 4*C*5). This object map was overlaid to the original scan (non‐median filter) and cementum was then segmented at 650 mg/cm^3^ only within the region traced in the median filter mask (Fig. 4*C*4,*C*5, mask, manual correction). Unmineralized tissues, ie, pulp and PDL, can be segmented semiautomatically using density boundaries (Fig. 4*B*5,*B*6,*C*2, fill void objects, dilate object, and walls/computer assist). It is essential to accurately describe segmentation strategies in publications as these can dramatically change results.

### Subregions

Further digital subdivision of tissues can be invaluable for dentoalveolar tissues, which exhibit a great degree of heterogeneity. Analyses can be targeted toward specific tissue anatomical subregions to detect changes that would otherwise be missed with whole tissue analyses. For example, subdivision of dentin volume allows comparison of crown versus root dentin or mesial versus distal molar roots. Alveolar bone is especially amenable to subdivision into anatomical regions and presents opportunities to address tissue‐specific scientific questions. For example, subdivisions may include separation of the mandible into basal versus alveolar bone, and alveolar bone can be subdivided into mesial versus distal, buccal versus lingual, or even tripartite division into cervical, middle, and apical alveolar bone (Fig. 4*D*1). Further, proximity can be used to define areas within a certain anatomical structure. In the example shown in Fig. 4*D*2, we labeled alveolar bone within 240 μm from the tooth root surface (Fig. 4*D*2, magenta) to indicate bone most involved with periodontal attachment (alveolar bone proper or ABP, as discussed in more detail below under Examples of Analyses: Alveolar Bone). Accordingly, the periodontal apparatus can be examined regionally or functionally, eg, the PDL, cementum, and alveolar bone can be subdivided into apical, middle, and coronal regions in parallel. Because subdivision can be specifically tailored to the experimental question, the approach must be described in detail when published, and a figure displaying subdivisions can be illustrative.^(^ ^69^ ^)^

In addition to determination of volume and density, thickness can be a useful measurement. Methods include taking multiple linear measurements and using software applications to measure average thickness over a given area.^(^ ^85^, ^86^, ^87^ ^)^ Single linear measurements are problematic because they are susceptible to high user bias and lack of reproducibility within and between users. Strategies to decrease bias include averaging multiple measurements, assigning a single user, calibrating multiple users, and ensuring reproducible orientations. In some software packages, the same formulas used to measure cortical thickness in long bones can be repurposed to measure thickness in dental tissues given that a hollow cylinder can be approximated by the tissue (eg, crown dentin). Some examples of thickness measurements are illustrated (Fig. 4*D*3).

## Examples of Analyses

To address specific research questions, application of appropriate scan settings (Fig. 2), regions of interest (Fig. 3), and segmentation techniques (Fig. 4), are required to provide appropriate data to test the hypothesis. In the next sections, we provide examples of how these recommended μCT practices were applied to real analyses of enamel, dentin, cementum, and alveolar bone in genetically edited mouse models.

### Enamel

**Fig 5:**
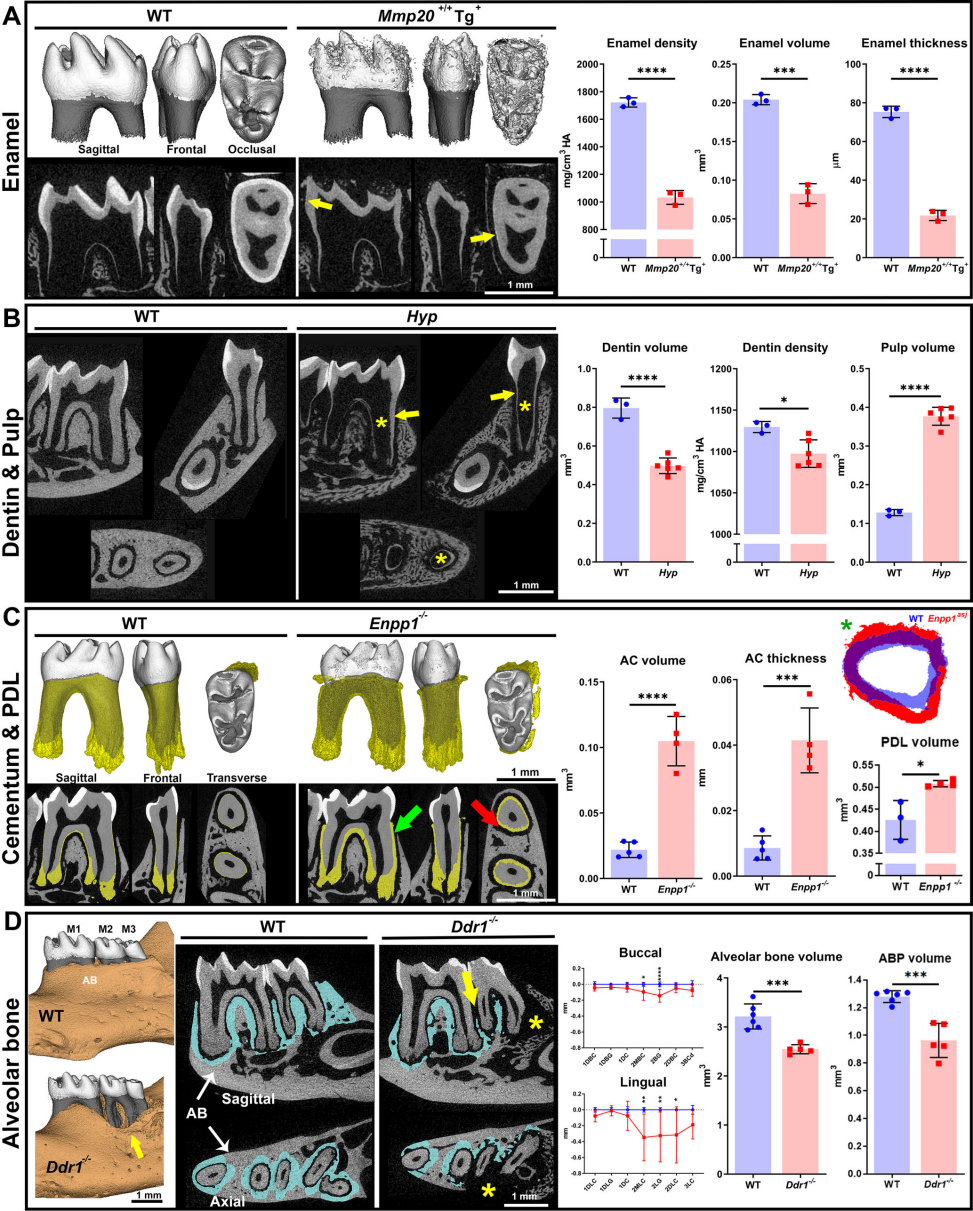
μCT analyses of dentoalveolar tissues in genetically engineered mouse models. Approaches described in this work are illustrated here for analysis of specific dentoalveolar tissues. (*A*) Enamel analysis was performed on *Mmp20* ^*+/+*^ *Tg* ^*+*^ overexpressing mice. This severe AI‐like phenotype is characterized by reduced enamel volume and density compared to WT. Enamel thickness was measured at lateral locations (yellow arrows) to avoid area of destruction on occlusal surfaces. (*B*) Dentin and pulp analyses were performed on the *Hyp* mutant mouse model of XLH. Compared to WT, Hyp mice exhibit decreased dentin volume and density (yellow arrows) corresponding with an increase in pulp volume (yellow *). (*C*) Cementum and PDL analyses were performed on the *Enpp1* mutant mouse model of GACI. *Enpp1* mutant mice feature dramatically increased AC volume and thickness compared to WT (green arrow). Increased PDL volume and thickness is also detected (red arrow and green *) in *Enpp1* mutant versus WT mice. (*D*) Alveolar bone analysis was performed on *Ddr1* mutant mice that feature periodontitis‐like bone loss at 9 months of age. Three μCT approaches were employed to quantify bone loss (yellow arrows and *). First, an adaptation of the classic linear measurement approach shows bone loss as increased distances up to ~0.4 mm from CEJ‐ABC at multiple locations on buccal and lingual aspects around *Ddr1* ^*−/−*^ versus WT molars. Locations are indicated by tooth^(^ ^1^, ^2^, ^3^ ^)^ and direction or feature (M, mesial; D; distal; B; buccal; L, lingual; C, cusp; G, groove), as described in Chavez and colleagues.^(^ ^44^ ^)^ Second, measurement of total alveolar bone loss around molars reveals 14% reduction in *Ddr1* ^*−/−*^ versus WT molars. Third, definition of ABP using a proximity technique (as described in the text and in Fig. 4) indicated a 30% reduction in ABP in *Ddr1* ^*−/−*^ versus WT molars. Statistical analysis was performed by independent samples *t* test; *p < .05; **p < .01; ***p < .001; ****p < .0001. (*A*) Reproduced and adapted with permission from Shin and colleagues.^(^ ^46^ ^)^ (*B*) Reproduced and adapted with permission from Zhang and colleagues.^(^ ^49^ ^)^ (*C*) Reproduced and adapted from Thumbigere‐Math and colleagues.^(^ ^23^ ^)^ (*D*) Reproduced and adapted from Chavez and colleagues.^(^ ^44^ ^)^ ABP = alveolar bone proper; AC = acellular cementum; AI = amelogenesis imperfecta; CEJ‐ABC = cementum‐enamel junction to alveolar bone crest; GACI = generalized arterial calcification in infancy; PDL = periodontal ligament; XLH = X‐linked hypophosphatasia.

Optimal evaluation of enamel depends on proper thresholding to avoid overestimating or underestimating enamel volume (Fig. 4*A*1–*A*3). In mouse studies where molars are erupted and amelogenesis has been completed, we typically threshold enamel at >1600 mg HA/cm^3^.^(^ ^44^, ^45^, ^48^, ^49^ ^)^ After thresholding and confirming segmentation of enamel from dentin, enamel volume and average mineral density are easily calculated. Although enamel is typically the most straightforward tissue to threshold and segment, severe defects create challenges for analysis; eg, when enamel and dentin densities become close or overlapping, when the enamel structure is severely disrupted (as in the example in Fig. 5A), or when enamel is too unstable to remain attached (also as in Fig. 5A).

Several mouse models of amelogenesis imperfecta (AI), an inherited disease affecting enamel formation, have been created and evaluated by μCT.^(^ ^9^, ^88^, ^89^ ^)^ Mice genetically ablated for matrix metalloproteinase 20 (MMP20) phenocopy a form of AI with hypomaturation defects that reflect secretory stage defects in amelogenesis.^(^ ^90^ ^)^ In studies analyzing the functions of MMP20, mice overexpressing this metalloproteinase (*Mmp20* ^*+/+*^ *Tg* ^*+*^) exhibited severe enamel defects marked by a patchy, thin, poorly defined enamel layer that delaminated from dentin (Fig. 5*A*).^(^ ^46^ ^)^ Molar enamel volume was decreased ~60% and mineral density reduced ~40%. Because enamel on the cusp regions of *Mmp20* ^*+/+*^ *Tg* ^*+*^ mice appeared to include ectopic blebs and flaking dentin, it was difficult to confidently define enamel borders and estimate thickness. In this scenario, we opted to measure enamel thickness at lateral locations on the first molar, where it would be less likely to break due to occlusal forces. Molar enamel thickness was calculated using cortical bone algorithms for the most median 25 axial slices (150 μm), as measured from the cementum‐enamel junction to the highest cusp tip. This approach revealed ~70% decrease in enamel thickness in *Mmp20* ^*+/+*^ *Tg* ^*+*^ versus control mice molars.

### Dentin and pulp

As the mineralized tissue composing the bulk of the tooth, dentin has been frequently analyzed by μCT. Although nonmineralized dental pulp is not directly analyzed by μCT, morphometric inferences can be made because it is enclosed by dentin. In mouse studies where molars are erupted and primary dentin formation is established, we typically threshold dentin at >650 mg HA/cm^3^.^(^ ^13^, ^15^, ^91^, ^92^ ^)^ After thresholding and confirming segmentation of dentin from enamel and bone, dentin volume and average mineral density can be calculated. Dentin and cementum volumes or densities are often reported as one collective measurement because of similar densities and proximity (though this volume is sometimes incorrectly identified simply as “dentin” in publications). This may result in misrepresentation of root dentin changes because alterations in cementum would also contribute to combined root tissue measurements. Additional μCT measurements or combination with histological or other approaches can clarify the situation. Subdivision can be useful in μCT analyses of dentin and pulp, eg, separating crown and root dentin for analysis, and this can be done by identifying the CEJ to demarcate crown versus root tissues.

Several inherited disorders affect dentinogenesis, including dentinogenesis imperfecta/dentin dysplasia, caused by mutations in dentin sialophosphoprotein (*DSPP*). Other mineralization disorders may affect dentin, including multiple forms of osteogenesis imperfecta (OI), hypophosphatasia (HPP), and genetic/congenital forms of hypophosphatemic rickets.^(^ ^48^, ^93^, ^94^, ^95^, ^96^ ^)^ *Hyp* mutant mice featuring mutations in phosphate‐regulating endopeptidase homolog X‐linked gene (*Phex*) represent a mouse model of X‐linked hypophosphatemia (XLH). In a study to define the precise dentoalveolar pathology associated with XLH, *Hyp* mice were found to feature severe mineralization disorders in multiple dentoalveolar tissues (Fig. 5*B*), notably expanded pulp chambers and dentin defects.^(^ ^49^ ^)^ Molar dentin volume was decreased ~30% to 40%, whereas dentin mineral density was decreased ~4% in *Hyp* versus control mice. Conversely, pulp volume was increased ~300% in *Hyp* mice compared to WT. These dentin measurements included the entire molar after segmenting out enamel and pulp space; therefore, they included a small contribution from cementum. In the case of *Hyp* mice, the dentin defects were so severe, and cementum was so reduced and/or hypomineralized, that there was no practical alternative but to calculate measurements for combined dentin/cementum, and this was noted in the Materials and Methods and Results sections in Zhang and colleagues,^(^ ^49^ ^)^ and explored further by other approaches, including histology, histomorphometry, scanning electron microscopy (SEM), and nanoindentation.

### Cementum and PDL

The cementum layer is diminutive in mice, particularly the acellular cementum on the cervical region of the root. To date, very few studies have used μCT to analyze either acellular or cellular cementum, therefore cementum volume, thickness, and mineral density have not been routinely included in publications. This is in spite of the fact that acellular cementum is critical for tooth attachment and cellular cementum comprises a significant portion of root volume. The primary reason for this is that segmentation of cementum from dentin presents several technical challenges not easily overcome. In histological slides, both acellular and cellular cementum are clearly identifiable from dentin based on selective staining (eg, H&E, toluidine blue, or picrosirius red stain viewed under polarized light, where cementum and dentin exhibit different organization and orientation of collagen fibers).^(^ ^97^ ^)^ In order to accurately define cementum by μCT, scan parameters must be sensitive enough to take advantage of the morphological, organizational, and relatively small density differences between cementum and dentin (as outlined above and in Fig. 4). As with pulp, nonmineralized PDL is not usually analyzed by μCT, but as an essential component of the periodontal complex that can adapt to and reflect changes in cementum. Analysis of PDL volume or thickness can be achieved after segmenting the adjacent hard tissues.

We have optimized scan parameters and analytical approaches for murine cementum. Parameters judged the most optimal for cellular cementum were 0.5‐mm Al filter, 70 kVp, 6 μm voxel size, and 900 ms integration time. For acellular cementum, mouse molars were dissected from the mandible and scanned in a smaller diameter sample holder (Scanco 6.0 mm) with settings of 0.5‐mm Al filter, 70 kVp, 2 μm voxel size, and 1200 ms integration time. In Fig. 4*C*, we show application of median filters and masks to segment cementum, and these techniques have been integrated in our studies.^(^ ^23^, ^45^, ^47^ ^)^

In a mouse model of generalized arterial calcification in infancy (GACI), mice genetically ablated for ectonucleotide pyrophosphatase phosphodiesterase 1 (*Enpp1* ^*−/−*^) featured ectopic calcification due to reduced levels of mineralization inhibitor, inorganic pyrophosphate (PP_i_). Compared to controls, *Enpp1* ^*−/−*^ mice featured dramatically increased cementum formation (Fig. 5*C*).^(^ ^23^, ^45^ ^)^ Acellular and cellular regions were defined by examination of μCT and histology, identifying cellular cementum in the apical one third of the root and acellular cementum in the cervical two thirds of the root. With this separation, *Enpp1* ^*−/−*^ mice exhibited a 500% increase in acellular cementum volume, whereas cellular cementum increased ~50%, compared to controls. We further evaluated the PDL space for alterations as a result of cementum expansion.^(^ ^47^ ^)^ PDL was segmented using a combination of semi‐automated and manual functions (see Fig. 4*B*, dilate object, walls). When PDL volumes from WT and *Enpp1* ^*−/−*^ mice were overlaid, we noted that PDL width was maintained and in fact volume was increased in *Enpp1* ^*−/−*^ compared to WT mice (Fig. 5*C*).

### Alveolar bone

Alveolar bone in rodents is studied for many reasons, including to advance our understanding of inherited and acquired bone disorders, therapies to increase bone quality or quantity, orthodontic tooth movement, and the aging process. Prescribed approaches for μCT analysis of long bones are well established.^(^ ^1^ ^)^ Using current image analysis software packages, detailed quantitative data can be generated, including bone volumes, two dimensional measurements (eg, cross‐sectional areas), and characteristics specific to different regions of bone (eg, cortical porosity, trabecular thickness, and periosteal perimeter). Unlike long bones, alveolar bone does not feature easily identifiable regions that are primarily trabecular (like the distal portion of a femur) or cortical (like the midshaft of a long bone). Instead, the alveolar bone is composed of a dense layer of cortical bone with an inner trabecular network of bone in some locations. Consequently, μCT analysis of rodent alveolar bone has been applied with a wide variety of approaches.^(^ ^11^, ^44^, ^98^ ^)^ These inconsistent approaches can be attributed to attempts to account for the heterogeneity of bone structure, organization, and function in the mandible. Earlier, we outlined several ways to analyze alveolar bone (eg, Figs. 4*A*–*D* and 5*D*), including linear measurements, volumetric measurements, and region‐specific measurements that can be tailored to specific scientific questions. Additionally, measurements such as trabecular connectivity degree, structure model index, degree of anisotropy, and cortical minimum moment of inertia can be obtained, which can be used in finite elements analysis. In all cases, ROIs must be carefully defined, and limitations should be considered and discussed.

Periodontal diseases are among the most prevalent on earth, causing cementum, PDL, and alveolar bone destruction and tooth loss, significantly affecting oral and overall health and quality of life.^(^ ^99^, ^100^, ^101^ ^)^ There is great utility in μCT to analyze periodontal pathology, repair, and regeneration. In a study to define the dentoalveolar functions of discoidin domain receptor 1 (DDR1), a collagen receptor tyrosine kinase that regulates cell functions and collagen fibrillogenesis and mineralization, mice genetically ablated for Ddr1 (*Ddr1* ^*−/−*^) exhibited severe alveolar bone loss with age (Fig. 5*D*).^(^ ^44^ ^)^ In that study, we employed μCT in three ways to quantify alveolar bone loss in this model: linear measurements, total alveolar bone volume, and a novel approach to measure “alveolar bone proper” (ABP) immediately adjacent to molar roots. In a modified method of the traditional caliper based of CEJ to alveolar bone crest (CEJ‐ABC) linear measurement,^(^ ^102^ ^)^ there was significant vertical bone loss up to 0.4 mm at five locations around *Ddr1* ^*−/−*^ molars. In a 3D volumetric analysis of alveolar bone surrounding all three mandibular molars, a 14% reduction in bone volume was detected in *Ddr1* ^*−/−*^ versus WT mice. To better approximate the actual loss of attachment that accompanies periodontal disease, we defined ABP as bone within 240 μm of the tooth root, as measured radially from tooth root surfaces to include buccal, lingual, radicular, and interproximal alveolar bone (Fig. 4*D*2). ABP, also called bundle bone in humans, corresponds to the lamina dura of dental radiographs and includes a high density of Sharpey's fibers that provide continuity between tooth‐PDL‐alveolar bone tissues.^(^ ^34^ ^)^ *Ddr1* ^*−/−*^ mice exhibited 30% reduction in ABP versus WT. Although μCT can approximate linear measurements of alveolar bone loss, 3D measurement of total alveolar bone better approximates volumetric changes. A strategy like that described for ABP increases sensitivity to detect bone loss even further, putting the focus on the functionally important bundle bone that anchors PDL fibers.

### Artifacts in scans of dentoalveolar tissues

There are several circumstances where μCT scanning artifacts may be created by appliances or materials, including dental implants or springs, wires, and composite materials used for orthodontic tooth movement (OTM) experiments.^(^ ^47^ ^)^ The scan optimization recommendations in this paper were optimized for biologic ranges in density; however, these materials typically have significantly higher densities than oral mineralized tissues. This situation is particularly difficult to correct because one approach to reduce these artifacts would be to increase voltage; however, this will simultaneously reduce quality of biologically relevant densities. Dual‐energy and multi‐energy X‐ray tomography are technologies that would potentially reduce this problem, as well as provide many other advantages in the segmentation of biological tissues. These techniques are currently being developed, and clinical applications are emerging.^(^ ^103^ ^)^ Dual‐energy quantitative X‐ray tomography, in which a dual‐energy X‐ray source is combined with the resolution of individual photon energy in the detector, is currently being developed and has an even greater potential in resolving materials that feature disparate densities within one sample.^(^ ^104^ ^)^ However, practical uses of these technologies are still under development. Alternatively, imaging processing algorithms have been proposed to reduce metal and other artifacts in CT scans,^(^ ^92^, ^105^, ^106^ ^)^ although, to our knowledge, these methods have been primarily implemented for CT, and few studies have translated them to μCT analysis.^(^ ^91^ ^)^ Because there may not be a way to optimally prevent or ameliorate artifacts, care should be taken in analysis and interpretation, and limitations should be discussed in publications.

## Summary of Guidelines

μCT has become an essential tool for analysis of mineralized tissues in mouse models; however, analyses of murine dentoalveolar tissues present unique challenges. Because of these inherent challenges, important considerations must be taken. Based on our optimization and application of strategies in dentoalveolar studies, we provide a list of recommendations for inclusion in studies utilizing μCT.

- Scanning parameters should be optimized for analysis of dentoalveolar tissues, or at least the target tissue(s) in the study, such that results are not tainted by artifacts or poor image acquisition, leading to spurious results. Parameters should be listed in the methods section, including at a minimum the make and model of scanner, the filter, voltage, and voxel size (resolution). Integration time has not been widely reported in studies, although we found this setting useful for scan optimization.
- Calibration to standards should be addressed; eg, if and how samples underwent calibration, and whether absolute or relative units were used in quantitative data.
- Orientation of samples should be described using appropriate anatomical landmarks.
- ROI should be carefully defined and/or shown by a figure.
- Segmentation techniques should be detailed in description of methods. Details should include thresholds applied, use of automated or semi‐automated approaches, and application of manual corrections. If tissues are subdivided for analysis, this must be carefully described, possibly with inclusion of images depicting subregions.
- Analysis software should be cited, and digital tools used for segmentation should be listed.

Our goals for this review parallel those outlined by Bouxsein and colleagues^(^ ^1^ ^)^ and Vardelis and Salmon^(^ ^16^ ^)^ in their indispensable reports. Specifically, this review is not meant to dictate specific approaches for assessing dentoalveolar tissues in mouse models, but rather to promote increased transparency and reproducibility, encourage best practices, and provide a basic framework that can be adapted to apply μCT analysis to dentoalveolar tissues. The methods and strategies described here for mouse dentoalveolar tissues may be extrapolated to dentoalveolar tissues of larger animals or to other skeletal regions. This requires understanding the use of X‐ray scanning instruments across length‐scales that are sensitive to space, density, and fields of view, but can be accomplished using methods described here.

## Disclosures

The authors declare that they have no known competing financial interests or personal relationships that could have appeared to influence the work reported in this manuscript.

## AUTHOR CONTRIBUTIONS

**Michael Chavez:** Conceptualization; formal analysis; investigation; methodology; writing‐original draft; writing‐review & editing. **Emily Chu:** Conceptualization; formal analysis; funding acquisition; investigation; methodology; writing‐original draft; writing‐review & editing. **Vardit Kram:** Investigation; methodology; writing‐original draft; writing‐review & editing. **Luis de Castro:** Investigation; methodology; writing‐original draft; writing‐review & editing. **Martha Somerman:** Conceptualization; supervision; writing‐original draft; writing‐review & editing. **Brian Foster:** Conceptualization; funding acquisition; supervision; writing‐original draft; writing‐review & editing.

### PEER REVIEW

The peer review history for this article is available at https://publons.com/publon/10.1002/jbm4.10474.

## References

[1] Bouxsein ML , Boyd SK , Christiansen BA , Guldberg RE , Jepsen KJ , Muller R . Guidelines for assessment of bone microstructure in rodents using micro‐computed tomography. J Bone Miner Res. 2010;25(7):1468‐1486. 20533309 10.1002/jbmr.141

[2] Clark DP , Badea CT . Micro‐CT of rodents: state‐of‐the‐art and future perspectives. Phys Med. 2014;30(6):619‐634. 24974176 10.1016/j.ejmp.2014.05.011 PMC4138257

[3] Faot F , Chatterjee M , de Camargos GV , Duyck J , Vandamme K . Micro‐CT analysis of the rodent jaw bone micro‐architecture: a systematic review. Bone Rep. 2015;2:14‐24. 28525530 10.1016/j.bonr.2014.10.005 PMC5365162

[4] van't Hof RJ , Dall'Ara E . Analysis of bone architecture in rodents using micro‐computed tomography. Methods Mol Biol. 2019;1914:507‐531. 30729484 10.1007/978-1-4939-8997-3_28

[5] Campbell GM , Sophocleous A . Quantitative analysis of bone and soft tissue by micro‐computed tomography: applications to ex vivo and in vivo studies. Bonekey Rep. 2014;3:564. 25184037 10.1038/bonekey.2014.59 PMC4140449

[6] Vasquez SX , Shah N , Hoberman AM . Small animal imaging and examination by micro‐CT. Methods Mol Biol. 2013;947:223‐231. 23138908 10.1007/978-1-62703-131-8_18

[7] Muller R , Van Campenhout H , Van Damme B , et al. Morphometric analysis of human bone biopsies: a quantitative structural comparison of histological sections and micro‐computed tomography. Bone. 1998;23(1):59‐66. 9662131 10.1016/s8756-3282(98)00068-4

[8] Mashiatulla M , Ross RD , Sumner DR . Validation of cortical bone mineral density distribution using micro‐computed tomography. Bone. 2017;99:53‐61. 28363808 10.1016/j.bone.2017.03.049 PMC5481667

[9] Schmitz JE , Teepe JD , Hu Y , Smith CE , Fajardo RJ , Chun YH . Estimating mineral changes in enamel formation by ashing/BSE and microCT. J Dent Res. 2014;93(3):256‐262. 24470541 10.1177/0022034513520548 PMC3929980

[10] Tsutsumi T , Kajiya H , Tsuzuki T , Goto KT , Okabe K , Takahashi Y . Micro‐computed tomography for evaluating alveolar bone resorption induced by hyperocclusion. J Prosthodont Res. 2018;62(3):298‐302. 29241945 10.1016/j.jpor.2017.11.004

[11] Park CH , Abramson ZR , Taba M Jr , et al. Three‐dimensional micro‐computed tomographic imaging of alveolar bone in experimental bone loss or repair. J Periodontol. 2007;78(2):273‐281. 17274716 10.1902/jop.2007.060252 PMC2581750

[12] Xu X , Zhou J , Yang F , Wei S , Dai H . Using micro‐computed tomography to evaluate the dynamics of orthodontically induced root resorption repair in a rat model. PLoS One. 2016;11(3):e0150135. 26930605 10.1371/journal.pone.0150135 PMC4773112

[13] Chatterjee M , Faot F , Correa C , Duyck J , Naert I , Vandamme K . A robust methodology for the quantitative assessment of the rat jawbone microstructure. Int J Oral Sci. 2017;9(2):87‐94. 28621323 10.1038/ijos.2017.11 PMC5518971

[14] Cox TC . Microcomputed tomography of craniofacial mineralized tissue: a practical user's guide to study planning and generating quality data. Bone. 2020;137:115408. 32407962 10.1016/j.bone.2020.115408

[15] Swain MV , Xue J . State of the art of micro‐CT applications in dental research. Int J Oral Sci. 2009;1(4):177‐188. 20690421 10.4248/IJOS09031 PMC3470105

[16] Verdelis K , Salmon P . Microcomputed tomography imaging in odontogenesis studies. Methods Mol Biol. 1922;2019:309‐324. 10.1007/978-1-4939-9012-2_28 30838586

[17] Dempster DW , Cosman F , Kurland ES , et al. Effects of daily treatment with parathyroid hormone on bone microarchitecture and turnover in patients with osteoporosis: a paired biopsy study. J Bone Miner Res. 2001;16(10):1846‐1853. 11585349 10.1359/jbmr.2001.16.10.1846

[18] du Plessis A , Broeckhoven C , Guelpa A , le Roux SG . Laboratory x‐ray micro‐computed tomography: a user guideline for biological samples. Gigascience. 2017;6(6):1‐11. 10.1093/gigascience/gix027 PMC5449646 28419369

[19] Laib A , Barou O , Vico L , Lafage‐Proust MH , Alexandre C , Rugsegger P . 3D micro‐computed tomography of trabecular and cortical bone architecture with application to a rat model of immobilisation osteoporosis. Med Biol Eng Comput. 2000;38(3):326‐332. 10912350 10.1007/BF02347054

[20] Vignero J , Marshall NW , Vande Velde G , Bliznakova K , Bosmans H . Translation from murine to human lung imaging using x‐ray dark field radiography: a simulation study. PLoS One. 2018;13(10):e0206302. 30372458 10.1371/journal.pone.0206302 PMC6205805

[21] Williams DK , Pinzon C , Huggins S , et al. Genetic engineering a large animal model of human hypophosphatasia in sheep. Sci Rep. 2018;8(1):16945. 30446691 10.1038/s41598-018-35079-y PMC6240114

[22] Ritman EL . Micro‐computed tomography‐current status and developments. Annu Rev Biomed Eng. 2004;6:185‐208. 15255767 10.1146/annurev.bioeng.6.040803.140130

[23] Thumbigere‐Math V , Alqadi A , Chalmers NI , et al. Hypercementosis associated with ENPP1 mutations and GACI. J Dent Res. 2018;97(4):432‐441. 29244957 10.1177/0022034517744773 PMC5863873

[24] Thesleff I , Aberg T . Molecular regulation of tooth development. Bone. 1999;25(1):123‐125. 10423036 10.1016/s8756-3282(99)00119-2

[25] Mitsiadis TA , Luder HU . Genetic basis for tooth malformations: from mice to men and back again. Clin Genet. 2011;80(4):319‐329. 21819395 10.1111/j.1399-0004.2011.01762.x

[26] Lesot H , Hovorakova M , Peterka M , Peterkova R . Three‐dimensional analysis of molar development in the mouse from the cap to bell stage. Aust Dent J. 2014;59(Suppl 1):81‐100. 10.1111/adj.12132 24495111

[27] Hu JC , Chun YH , Al Hazzazzi T , Simmer JP . Enamel formation and amelogenesis imperfecta. Cells Tissues Organs. 2007;186(1):78‐85. 17627121 10.1159/000102683

[28] Lacruz RS , Habelitz S , Wright JT , Paine ML . Dental enamel formation and implications for oral health and disease. Physiol Rev. 2017;97(3):939‐993. 28468833 10.1152/physrev.00030.2016 PMC6151498

[29] Fincham AG , Moradian‐Oldak J , Simmer JP . The structural biology of the developing dental enamel matrix. J Struct Biol. 1999;126(3):270‐299. 10441532 10.1006/jsbi.1999.4130

[30] Goldberg M , Kulkarni AB , Young M , Boskey A . Dentin: structure, composition and mineralization. Front Biosci (Elite Ed). 2011;3:711‐735. 21196346 10.2741/e281 PMC3360947

[31] Verdelis K , Lukashova L , Wright JT , et al. Maturational changes in dentin mineral properties. Bone. 2007;40(5):1399‐1407. 17289453 10.1016/j.bone.2006.12.061 PMC1913214

[32] Marshall GW Jr . Dentin: microstructure and characterization. Quintessence Int. 1993;24(9):606‐617. 8272499

[33] Foster BL , Popowics TE , Fong HK , Somerman MJ . Advances in defining regulators of cementum development and periodontal regeneration. Curr Top Dev Biol. 2007;78:47‐126. 17338915 10.1016/S0070-2153(06)78003-6

[34] Nanci A . Periodontium. Ten Cate's Oral Histology. 9th ed. Elsevier; 2017 pp 193‐217.

[35] Bosshardt DD . Are cementoblasts a subpopulation of osteoblasts or a unique phenotype? J Dent Res. 2005;84(5):390‐406. 15840773 10.1177/154405910508400501

[36] Sodek J , McKee MD . Molecular and cellular biology of alveolar bone. Periodontol 2000. 2000;24:99‐126. 11276877 10.1034/j.1600-0757.2000.2240106.x

[37] Boskey AL . Bone composition: relationship to bone fragility and antiosteoporotic drug effects. Bonekey Rep. 2013;2:447. 24501681 10.1038/bonekey.2013.181 PMC3909232

[38] Beertsen W , McCulloch CA , Sodek J . The periodontal ligament: a unique, multifunctional connective tissue. Periodontol 2000. 1997;13:20‐40. 9567922 10.1111/j.1600-0757.1997.tb00094.x

[39] Stauber M , Muller R . Micro‐computed tomography: a method for the non‐destructive evaluation of the three‐dimensional structure of biological specimens. Methods Mol Biol. 2008;455:273‐292. 18463825 10.1007/978-1-59745-104-8_19

[40] Boerckel JD , Mason DE , McDermott AM , Alsberg E . Microcomputed tomography: approaches and applications in bioengineering. Stem Cell Res Ther. 2014;5(6):144. 25689288 10.1186/scrt534 PMC4290379

[41] Hupfer M , Nowak T , Brauweiler R , Eisa F , Kalender WA . Spectral optimization for micro‐CT. Med Phys. 2012;39(6):3229‐3239. 22755706 10.1118/1.4718575

[42] Schambach SJ , Bag S , Schilling L , Groden C , Brockmann MA . Application of micro‐CT in small animal imaging. Methods. 2010;50(1):2‐13. 19706326 10.1016/j.ymeth.2009.08.007

[43] Behrooz A , Kask P , Meganck J , Kempner J . Automated quantitative bone analysis in in vivo X‐ray micro‐computed tomography. IEEE Trans Med Imaging. 2017;36(9):1955‐1965. 28600241 10.1109/TMI.2017.2712571

[44] Chavez MB , Kolli TN , Tan MH , et al. Loss of discoidin domain receptor 1 predisposes mice to periodontal breakdown. J Dent Res. 2019;98(13):1521‐1531. 31610730 10.1177/0022034519881136 PMC6873285

[45] Chu EY , Vo TD , Chavez MB , et al. Genetic and pharmacologic modulation of cementogenesis via pyrophosphate regulators. Bone. 2020;136:115329. 10.1016/j.bone.2020.115329. Epub 2020 Mar 26. PMC7482720 32224162

[46] Shin M , Chavez MB , Ikeda A , Foster BL , Bartlett JD . MMP20 overexpression disrupts molar ameloblast polarity and migration. J Dent Res. 2018;97(7):820‐827. 29481294 10.1177/0022034518758657 PMC6728588

[47] Wolf M , Ao M , Chavez MB , et al. Reduced orthodontic tooth movement in Enpp1 mutant mice with hypercementosis. J Dent Res. 2018;97(8):937‐945. 29533727 10.1177/0022034518759295 PMC6728553

[48] Xu H , Lenhart SA , Chu EY , et al. Dental and craniofacial defects in the Crtap(^−/−^) mouse model of osteogenesis imperfecta type VII. Dev Dyn. 2020;249(7):884‐897. 32133710 10.1002/dvdy.166 PMC7727892

[49] Zhang H , Chavez MB , Kolli TN , et al. Dentoalveolar defects in the Hyp mouse model of X‐linked hypophosphatemia. J Dent Res. 2020;99(4):419‐428. 31977267 10.1177/0022034520901719 PMC7088204

[50] Christiansen BA . Effect of micro‐computed tomography voxel size and segmentation method on trabecular bone microstructure measures in mice. Bone Rep. 2016;5:136‐140. 27430011 10.1016/j.bonr.2016.05.006 PMC4926804

[51] Oliviero S , Lu Y , Viceconti M , Dall'Ara E . Effect of integration time on the morphometric, densitometric and mechanical properties of the mouse tibia. J Biomech. 2017;65:203‐211. 29126603 10.1016/j.jbiomech.2017.10.026

[52] Alyahya A , Alqareer A , Swain M . Microcomputed tomography calibration using polymers and minerals for enamel mineral content quantitation. Med Princ Pract. 2019;28(3):247‐255. 30820021 10.1159/000499186 PMC6597939

[53] Zou W , Gao J , Jones AS , Hunter N , Swain MV . Characterization of a novel calibration method for mineral density determination of dentine by X‐ray micro‐tomography. Analyst. 2009;134(1):72‐79. 19082177 10.1039/b806884d

[54] Nazarian A , Snyder BD , Zurakowski D , Muller R . Quantitative micro‐computed tomography: a non‐invasive method to assess equivalent bone mineral density. Bone. 2008;43(2):302‐311. 18539557 10.1016/j.bone.2008.04.009

[55] Burghardt AJ , Kazakia GJ , Laib A , Majumdar S . Quantitative assessment of bone tissue mineralization with polychromatic micro‐computed tomography. Calcif Tissue Int. 2008;83(2):129‐138. 18685797 10.1007/s00223-008-9158-x PMC2801565

[56] Schweizer S , Hattendorf B , Schneider P , et al. Preparation and characterization of calibration standards for bone density determination by micro‐computed tomography. Analyst. 2007;132(10):1040‐1045. 17893808 10.1039/b703220j

[57] Entezari V , Vartanians V , Zurakowski D , et al. Further improvements on the factors affecting bone mineral density measured by quantitative micro‐computed tomography. Bone. 2012;50(3):611‐618. 22044640 10.1016/j.bone.2011.10.004

[58] Sekhon K , Kazakia GJ , Burghardt AJ , Hermannsson B , Majumdar S . Accuracy of volumetric bone mineral density measurement in high‐resolution peripheral quantitative computed tomography. Bone. 2009;45(3):473‐479. 19501201 10.1016/j.bone.2009.05.023 PMC4454742

[59] Fajardo RJ , Cory E , Patel ND , et al. Specimen size and porosity can introduce error into microCT‐based tissue mineral density measurements. Bone. 2009;44(1):176‐184. 18822398 10.1016/j.bone.2008.08.118 PMC4286574

[60] Cann CE . Quantitative CT for determination of bone mineral density: a review. Radiology. 1988;166(2):509‐522. 3275985 10.1148/radiology.166.2.3275985

[61] Johnston SM , Johnson GA , Badea CT . Geometric calibration for a dual tube/detector micro‐CT system. Med Phys. 2008;35(5):1820‐1829. 18561657 10.1118/1.2900000 PMC2809730

[62] Cho Y , Moseley DJ , Siewerdsen JH , Jaffray DA . Accurate technique for complete geometric calibration of cone‐beam computed tomography systems. Med Phys. 2005;32(4):968‐983. 15895580 10.1118/1.1869652

[63] Ren L , Ghani MU , Wu D , et al. The impact of spectral filtration on image quality in micro‐CT system. J Appl Clin Med Phys. 2016;17(1):301‐315. 26894340 10.1120/jacmp.v17i1.5714 PMC4762071

[64] Hipsley CA , Aguilar R , Black JR , Hocknull SA . High‐throughput microCT scanning of small specimens: preparation, packing, parameters and post‐processing. Sci Rep. 2020;10(1):13863. 32807929 10.1038/s41598-020-70970-7 PMC7431592

[65] Rueckel J , Stockmar M , Pfeiffer F , Herzen J . Spatial resolution characterization of a X‐ray microCT system. Appl Radiat Isot. 2014;94:230‐234. 25233529 10.1016/j.apradiso.2014.08.014

[66] Ostertag A , Peyrin F , Gouttenoire PJ , et al. Multiscale and multimodality computed tomography for cortical bone analysis. Phys Med Biol. 2016;61(24):8553‐8576. 27845939 10.1088/0031-9155/61/24/8553

[67] Peyrin F , Salome M , Cloetens P , Laval‐Jeantet AM , Ritman E , Ruegsegger P . Micro‐CT examinations of trabecular bone samples at different resolutions: 14, 7 and 2 micron level. Technol Health Care. 1998;6(5–6):391‐401. 10100941

[68] Kalatzis‐Sousa NG , Spin‐Neto R , Wenzel A , Tanomaru‐Filho M , Faria G . Use of micro‐computed tomography for the assessment of periapical lesions in small rodents: a systematic review. Int Endod J. 2017;50(4):352‐366. 26992821 10.1111/iej.12633

[69] Foster BL , Ao M , Salmon CR , et al. Osteopontin regulates dentin and alveolar bone development and mineralization. Bone. 2018;107:196‐207. 29313816 10.1016/j.bone.2017.12.004 PMC5803363

[70] Taut AD , Jin Q , Chung JH , et al. Sclerostin antibody stimulates bone regeneration after experimental periodontitis. J Bone Miner Res. 2013;28(11):2347‐2356. 23712325 10.1002/jbmr.1984

[71] Ghosh P , Stabley JN , Behnke BJ , Allen MR , Delp MD . Effects of spaceflight on the murine mandible: possible factors mediating skeletal changes in non‐weight bearing bones of the head. Bone. 2016;83:156‐161. 26545335 10.1016/j.bone.2015.11.001

[72] Pal A , Chen L , Yang L , et al. Micro‐anatomical responses in periodontal complexes of mice to calibrated orthodontic forces on the crown. Orthod Craniofac Res. 2017;20(Suppl 1):100‐105. 28643923 10.1111/ocr.12172

[73] Cantley MD , Bartold PM , Marino V , et al. The use of live‐animal micro‐computed tomography to determine the effect of a novel phospholipase A2 inhibitor on alveolar bone loss in an in vivo mouse model of periodontitis. J Periodontal Res. 2009;44(3):317‐322. 19462493 10.1111/j.1600-0765.2008.01132.x

[74] Sasaki H , Furusho H , Rider DB , et al. Endodontic infection‐induced inflammation resembling osteomyelitis of the jaws in toll‐like receptor 2/interleukin 10 double‐knockout mice. J Endod. 2019;45(2):181‐188. 30711175 10.1016/j.joen.2018.10.007 PMC6364571

[75] AlShwaimi E , Berggreen E , Furusho H , et al. IL‐17 receptor A signaling is protective in infection‐stimulated periapical bone destruction. J Immunol. 2013;191(4):1785‐1791. 23863904 10.4049/jimmunol.1202194 PMC3767040

[76] Verdelis K , Szabo‐Rogers HL , Xu Y , et al. Accelerated enamel mineralization in Dspp mutant mice. Matrix Biol. 2016;52–54:246‐259. 10.1016/j.matbio.2016.01.003 PMC4875851 26780724

[77] Kono K , Tanikawa C , Yanagita T , Kamioka H , Yamashiro T . A novel method to detect 3D mandibular changes related to soft‐diet feeding. Front Physiol. 2017;8:567. 28855872 10.3389/fphys.2017.00567 PMC5557733

[78] Scherf H , Tilgner R . A new high‐resolution computed tomography (CT) segmentation method for trabecular bone architectural analysis. Am J Phys Anthropol. 2009;140(1):39‐51. 19280676 10.1002/ajpa.21033

[79] Djomehri SI , Candell S , Case T , et al. Mineral density volume gradients in normal and diseased human tissues. PLoS One. 2015;10(4):e0121611. 25856386 10.1371/journal.pone.0121611 PMC4391782

[80] Kramer K , Chavez MB , Tran AT , et al. Dental defects in the primary dentition associated with hypophosphatasia from biallelic ALPL mutations. Bone. 2021;143:115732. 10.1016/j.bone.2020.115732. Epub 2020 Nov 4. 33160095 PMC7769999

[81] Ao M , Chavez MB , Chu EY , et al. Overlapping functions of bone sialoprotein and pyrophosphate regulators in directing cementogenesis. Bone. 2017;105:134‐147. 28866368 10.1016/j.bone.2017.08.027 PMC5730356

[82] Gomez W , Sales E , Lopes RT , Pereira WC . A comparative study of automatic thresholding approaches for 3D x‐ray microtomography of trabecular bone. Med Phys. 2013;40(9):091903. 24007154 10.1118/1.4817235

[83] Bosshardt DD , Schroeder HE . Initial formation of cellular intrinsic fiber cementum in developing human teeth. A light‐ and electron‐microscopic study. Cell Tissue Res. 1992;267(2):321‐335. 1600564 10.1007/BF00302971

[84] Foster BL , Somerman MJ . Cementum. In McCauley LK , Somerman MJ , eds. Mineralized tissues in oral and craniofacial science: biological principles and clinical correlates. 1st ed. Wiley‐Blackwell; 2012 pp 169‐192.

[85] Delecourt C , Relier M , Touraine S , et al. Cartilage morphology assessed by high resolution micro‐computed tomography in non OA knees. Osteoarthritis Cartilage. 2016;24(3):567‐571. 26505662 10.1016/j.joca.2015.10.009

[86] Hostetler ZS , Stitzel JD , Weaver AA . Comparing rib cortical thickness measurements from computed tomography (CT) and micro‐CT. Comput Biol Med. 2019;111:103330. 31276944 10.1016/j.compbiomed.2019.103330 PMC6785990

[87] Olejniczak AJ , Grine FE . Assessment of the accuracy of dental enamel thickness measurements using microfocal X‐ray computed tomography. Anat Rec A Discov Mol Cell Evol Biol. 2006;288(3):263‐275. 16463379 10.1002/ar.a.20307

[88] Hu Y , Smith CE , Richardson AS , Bartlett JD , Hu JC , Simmer JP . MMP20, KLK4, and MMP20/KLK4 double null mice define roles for matrix proteases during dental enamel formation. Mol Genet Genomic Med. 2016;4(2):178‐196. 27066511 10.1002/mgg3.194 PMC4799876

[89] Nunez SM , Chun YP , Ganss B , et al. Maturation stage enamel malformations in Amtn and Klk4 null mice. Matrix Biol. 2016;52–54:219‐233. 10.1016/j.matbio.2015.11.007 PMC4875837 26620968

[90] Bartlett JD . Dental enamel development: proteinases and their enamel matrix substrates. ISRN Dent. 2013;2013:684607. 24159389 10.1155/2013/684607 PMC3789414

[91] Gabet Y , Bab I . A validated method for titanium implant anchorage analysis using microCT and biomechanical testing. Adv Tech Biol Med. 2016;4(3):1000180. 10.4172/2379-1764.1000180.

[92] Hegazy MA , Cho MH , Lee SY . A metal artifact reduction method for a dental CT based on adaptive local thresholding and prior image generation. Biomed Eng Online. 2016;15(1):119. 27814775 10.1186/s12938-016-0240-8 PMC5097357

[93] Foster BL , Kuss P , Yadav MC , et al. Conditional Alpl ablation phenocopies dental defects of hypophosphatasia. J Dent Res. 2017;96(1):81‐91. 27582029 10.1177/0022034516663633 PMC5347426

[94] Eimar H , Tamimi F , Retrouvey JM , Rauch F , Aubin JE , McKee MD . Craniofacial and dental defects in the Col1a1Jrt/+ mouse model of osteogenesis imperfecta. J Dent Res. 2016;95(7):761‐768. 26951553 10.1177/0022034516637045

[95] Fang PA , Verdelis K , Yang X , Lukashova L , Boskey AL , Beniash E . Ultrastructural organization of dentin in mice lacking dentin sialo‐phosphoprotein. Connect Tissue Res. 2014;55(Suppl 1):92‐96. 25158189 10.3109/03008207.2014.923861 PMC4338995

[96] Boskey AL , Verdelis K , Spevak L , et al. Mineral and matrix changes in Brtl/+ teeth provide insights into mineralization mechanisms. Biomed Res Int. 2013;2013:295812. 23802117 10.1155/2013/295812 PMC3681234

[97] Foster BL . Methods for studying tooth root cementum by light microscopy. Int J Oral Sci. 2012;4(3):119‐128. 22996273 10.1038/ijos.2012.57 PMC3464984

[98] Lee MM , Chu EY , El‐Abbadi MM , et al. Characterization of mandibular bone in a mouse model of chronic kidney disease. J Periodontol. 2010;81(2):300‐309. 20151810 10.1902/jop.2009.090379 PMC2862731

[99] Ferreira MC , Dias‐Pereira AC , Branco‐de‐Almeida LS , Martins CC , Paiva SM . Impact of periodontal disease on quality of life: a systematic review. J Periodontal Res. 2017;52(4):651‐665. 28177120 10.1111/jre.12436

[100] Eke PI , Dye BA , Wei L , et al. Update on prevalence of periodontitis in adults in the United States: NHANES 2009 to 2012. J Periodontol. 2015;86(5):611‐622. 25688694 10.1902/jop.2015.140520 PMC4460825

[101] Petersen PE , Ogawa H . The global burden of periodontal disease: towards integration with chronic disease prevention and control. Periodontol 2000. 2012;60(1):15‐39. 22909104 10.1111/j.1600-0757.2011.00425.x

[102] Abe T , Hajishengallis G . Optimization of the ligature‐induced periodontitis model in mice. J Immunol Methods. 2013;394(1–2):49‐54. 23672778 10.1016/j.jim.2013.05.002 PMC3707981

[103] McCollough CH , Leng S , Yu L , Fletcher JG . Dual‐ and multi‐energy CT: principles, technical approaches, and clinical applications. Radiology. 2015;276(3):637‐653. 26302388 10.1148/radiol.2015142631 PMC4557396

[104] Sellerer T , Ehn S , Mechlem K , et al. Quantitative dual‐energy micro‐CT with a photon‐counting detector for material science and non‐destructive testing. PLoS One. 2019;14(7):e0219659. 31314812 10.1371/journal.pone.0219659 PMC6636745

[105] Boas FE , Fleischmann D . Evaluation of two iterative techniques for reducing metal artifacts in computed tomography. Radiology. 2011;259(3):894‐902. 21357521 10.1148/radiol.11101782

[106] Huang X , Wang J , Tang F , Zhong T , Zhang Y . Metal artifact reduction on cervical CT images by deep residual learning. Biomed Eng Online. 2018;17(1):175. 30482231 10.1186/s12938-018-0609-y PMC6260559

